# Multi-Parametric Evaluation of a Novel Benzoylthiourea Derivative as a Combustion Modifier in Diesel–Ethanol Blends Under EGR Conditions

**DOI:** 10.3390/molecules31111910

**Published:** 2026-06-02

**Authors:** Sertaç Coşman

**Affiliations:** Department of Mechanical Engineering, Faculty of Engineering and Architecture, Burdur Mehmet Akif Ersoy University, Burdur 15200, Türkiye; scosman@mehmetakif.edu.tr

**Keywords:** benzoylthiourea, diesel engine, engine emissions, exhaust gas recirculation, fuel additive

## Abstract

This study reports the first synthesis and full spectroscopic characterization (FT-IR, ^1^H NMR, ^13^C NMR) of a novel benzoylthiourea-based compound 2-chloro-N-((2-hydroxy-4-nitrophenyl)carbamothioyl)benzamide (HNCB) and evaluates its behavior as a combustion-modifying additive in diesel–ethanol blends. Blends containing 50, 100, and 200 ppm HNCB were tested in a single-cylinder direct-injection compression ignition engine at five torque levels (0–24 Nm) and four Exhaust gas recirculation rates (0–30%) to assess combustion, performance, and emissions. Ethanol improved mixture formation and combustion stability, while HNCB, particularly at 100 ppm, provided the most favorable overall balance of combustion phasing, heat-release characteristics, and emission control. At 24 Nm and 0% exhaust gas recirculation, Diesel + Ethanol + HNCB (100 ppm) increased maximum cylinder pressure by 4.1% relative to diesel and reduced cyclic indicated mean effective pressure variability. The 50 ppm blend yielded the lowest specific fuel consumption, with reductions of up to 37% at partial loads and the highest brake thermal efficiency values under several exhaust gas recirculation conditions. Nitrogen oxides emissions decreased by up to 65–75%, whereas the 200 ppm blend increased hydrocarbon and soot at 30% exhaust gas recirculation. Overall, HNCB acted as an effective combustion modifier under the tested conditions.

## 1. Introduction

Rapid industrialization and rising global energy demand have intensified reliance on fossil fuels, leading to increased greenhouse gas emissions, particularly carbon dioxide (CO_2_), which accounts for nearly 60% of total atmospheric greenhouse gases [1]. Within this context, the transportation sector remains a major contributor, with road transport responsible for about one-fifth of the European Union’s total CO_2_ emissions, 75% of which arise from passenger vehicles. Despite recent marginal improvements, transportation is still the only major sector in the EU where emissions continue to increase, challenging long-term climate objectives [2]. In addition to climate impacts, fossil fuel use aggravates land, air, and water pollution, posing serious risks to environmental sustainability and human health. Coupled with accelerating resource depletion driven by population growth and overconsumption, this highlights the urgent need for sustainable energy strategies and a transition toward renewable systems [3]. Although internal combustion engines (ICEs) remain dominant in current mobility, their environmental footprint has accelerated interest in low-carbon and carbon-neutral fuels [4]. Clean energy carriers such as green hydrogen, biofuels, and synthetic fuels offer promising pathways. Carbon-free fuels, especially hydrogen, have also attracted growing attention as long-term alternatives for minimizing engine-related carbon emissions, because their combustion does not directly generate carbon dioxide. Nevertheless, issues related to fuel storage, distribution infrastructure, combustion characteristics, and the overall environmental footprint of production routes remain important barriers to large-scale practical adoption [5,6]. However, their benefits may be compromised by upstream emissions, particularly methane leakage during natural gas-based hydrogen production, which can offset climate gains if not adequately controlled [7,8,9]. Consequently, achieving long-term sustainability requires an integrated approach combining alternative fuels, advanced combustion technologies, and effective emission control policies [10].

Internal combustion engines (ICEs) are heat engines that generate mechanical power through fuel combustion within a confined chamber and have long formed the backbone of transportation and industrial power systems due to their high energy density and operational reliability. However, their extensive reliance on fossil fuels has raised growing environmental concerns, particularly related to greenhouse gas emissions and harmful air pollutants [11]. The combustion process in ICEs leads directly to the release of carbon dioxide along with regulated pollutants such as carbon monoxide (CO), hydrocarbons (HC), nitrogen oxides (NO_x_), and particulate matter (PM). In response, emission regulations targeting these pollutants have been progressively enforced since the 1960s, with increasingly stringent limits introduced in major markets. More recently, national and international policies have further emphasized reducing carbon emissions. As a result, ICEs are widely recognized as significant contributors to CO_2_ emissions in the transportation sector, making emission reduction a central focus of contemporary research [12,13,14]. Among the primary strategies explored is the adoption of sustainable fuel alternatives, including biofuels, hydrogen, and natural gas, as substitutes for conventional fossil-based fuels [15].

Recent promising alternative fuels include methanol, biogas, biodiesel, and hydrogen, which are widely considered viable substitutes for diesel in compression ignition (CI) engines and gasoline in spark ignition (SI) engines. In parallel, fuel additives have attracted growing attention due to their ability to simultaneously improve engine performance, enhance combustion efficiency, and reduce exhaust emissions [16,17]. As a cost-effective approach, additives ranging from oxygenated compounds to metal-oxide and nanoparticle formulations can promote improved fuel–air mixing, accelerate combustion kinetics, and suppress peak in-cylinder temperatures, leading to reductions in CO, HC, NO_x_, and particulate emissions [18,19,20,21]. Accordingly, numerous experimental and review studies have focused on the application of fuel additives in diesel engines. Koçyiğit et al. reported that ethanol–propolis blends reduced key combustion parameters and regulated emissions while increasing CO_2_ emissions, indicating improved oxidation efficiency [22]. Pullagura et al. demonstrated that TiO_2_ nanoparticles combined with dimethyl carbonate significantly reduced BSFC, CO, HC, and NO_x_ emissions while improving brake thermal efficiency at full load [23]. Appavu et al. highlighted the effectiveness of EGR in conjunction with fuel additives for NO_x_ control in biodiesel-fueled CI engines, reporting reductions up to 75% under optimized conditions [24]. Elkelawy et al. showed that commercial fuel additives markedly decreased CO, HC, soot, and NO_x_ emissions in both diesel and biodiesel-based systems [25,26]. Further studies have confirmed that nanoparticle-based additives enhance thermal efficiency and reduce fuel consumption, indicating improved combustion effectiveness [27,28,29,30]. Similar benefits, including reductions in HC, CO, NO_x_, and smoke emissions alongside gains in thermal efficiency, have been reported for various biodiesel–diesel–additive blends under different operating conditions [31]. Overall, the beneficial influence of fuel additives on diesel engine performance and emissions has been consistently demonstrated and remains an active area of research interest [32,33,34,35]. Nevertheless, the application of fuel additives is not without challenges. Depending on their chemical nature and dosage, additives may adversely affect fuel stability, atomization behavior, combustion consistency, deposit formation tendency, and long-term compatibility with fuel system components. These considerations highlight the importance of careful additive design, concentration optimization, and application-specific evaluation [36].

The exploration of chemically diverse fuel additives for internal combustion engine applications has gained increasing attention in efforts to achieve cleaner and more efficient energy conversion. Such additives are designed to improve combustion quality, enhance engine efficiency, and reduce harmful exhaust emissions. Among the various candidate compounds, thiourea and its structural derivatives have emerged as promising additives due to their distinctive physicochemical properties and potential roles in combustion enhancement and emission control. In particular, aroylthioureas represent an important class of organosulfur and organonitrogen compounds, distinguished by the coexistence of acyl and thiourea functional groups within a single molecular framework [37]. These compounds have been extensively studied for their diverse biological activities, including antimicrobial, antioxidant, antiviral, and enzyme inhibitory effects, highlighting their structural versatility and chemical reactivity [38,39,40]. Beyond biological applications, aroylthioureas serve as valuable intermediates in organic synthesis and catalysts in various chemical processes, owing to their strong hydrogen-bonding capability and metal-coordination behavior [41,42].

Beyond their pharmaceutical and synthetic applications, aroylthioureas have also attracted attention as potential fuel additives. Previous studies have demonstrated that sulfur- and nitrogen-containing compounds can enhance combustion efficiency and reduce toxic emissions when blended with diesel or gasoline fuels [43]. In this context, Keskin et al. investigated metal-based additives such as acetylferrocene and a palladium complex (PdL_2_) in biodiesel–diesel blends and reported substantial reductions in CO and particulate matter emissions, by approximately 60% and 51%, respectively, accompanied by decreases in sound pressure level and vibration intensity, highlighting the effectiveness of low-dose metal additives in improving combustion behavior [44]. In a related study, PdL_2_ and NiL_2_ complexes added at 50–100 ppm produced no significant changes in engine torque or brake power, while achieving notable reductions in CO, NO_x_, and smoke emissions, together with a measurable decrease in BSFC [45]. Gürü et al. examined nickel- and magnesium-based additives synthesized to improve biodiesel derived from waste animal fat and reported favorable modifications in key fuel properties, including reduced viscosity and pour point, while maintaining engine performance comparable to conventional diesel. Notably, the B20 blend exhibited a higher cetane number and ensured stable engine operation [46]. Bora et al. synthesized a MgO/MgSO_4_ nanocatalyst using thiourea in a solution combustion process and demonstrated that the resulting biodiesel fully met ASTM fuel standards, supporting its suitability for practical applications [47]. Additionally, urea/thiourea complexation has been shown to enhance the octane number of FCC gasoline by selectively removing n-alkanes, thereby improving gasoline quality [48]. Similarly, the application of a benzoylthiourea derivative as a gasoline additive resulted in thermal efficiency improvements of up to 25%, along with approximately 20% reductions in specific fuel consumption and noticeable decreases (up to 23%) in hydrocarbon emissions [49]. Collectively, these studies underscore the potential of thiourea-based compounds as effective fuel additives and motivate continued efforts toward the structural optimization of such additives for improved engine performance and emission control.

In response to the growing interest in heteroatom-containing organic compounds as multifunctional fuel additives, this study reports the synthesis and engine application of a novel sulfur-based benzoylthiourea derivative for diesel engines. A novel compound identified as 2-chloro-N-((2-hydroxy-4-nitrophenyl)carbamothioyl)benzamide (HNCB) was synthesized for the first time and structurally confirmed using FT-IR, ^1^H-NMR, and ^13^C-NMR analyses. The synthesized additive was evaluated at different concentrations in a direct-injection single-cylinder compression ignition engine under varying load and exhaust gas recirculation (EGR) conditions. The influence of HNCB on key engine performance parameters, including specific fuel consumption, thermal efficiency, exhaust gas temperature, and air–fuel ratio, was systematically examined. In addition, comprehensive emission analyses were performed, focusing on CO_2_, CO, HC, NO_x_, and O_2_ emissions to assess their potential for promoting cleaner combustion. Unlike conventional additives, HNCB integrates both nitrogen and sulfur functionalities within its molecular structure, enabling modification of combustion-related behavior under diluted intake conditions. The findings provide new insights into the applicability of structurally tailored benzoylthiourea derivatives as multifunctional fuel additives for improving diesel engine efficiency and emission characteristics.

## 2. Materials and Methods

### 2.1. Materials

2-chloro-N-((2-hydroxy-4-nitrophenyl)carbamothioyl)benzamide (HNCB) was sourced from Faculty of Arts and Science, Department of Chemistry, Burdur Mehmet Akif Ersoy University. All chemicals and solvents used for the synthesis of the thiourea derivative were of analytical grade and used as received without further purification. Potassium thiocyanate (Merck, Darmstadt, Germany), 2-amino-5-nitrophenol (Sigma-Aldrich, St. Louis, MO, USA), 2-chlorobenzoyl chloride (Sigma-Aldrich, St. Louis, MO, USA), ethanol (Sigma-Aldrich, St. Louis, MO, USA), dichloromethane (Merck, Darmstadt, Germany), and hydrochloric acid (Merck, Darmstadt, Germany) were purchased from commercial suppliers.

### 2.2. Instrumentation

The FT-IR spectrum of the synthesized thiourea derivative was recorded in the range of 4000–400 cm^−1^ using a Shimadzu IRTracer-100 FT-IR spectrophotometer (Shimadzu Corporation, Kyoto, Japan). The ^1^H and ^13^C NMR spectra were acquired on a Bruker Avance III 400 MHz NMR spectrometer, with DMSO-*d*_6_ (Sigma-Aldrich, St. Louis, MO, USA) as the solvent and tetramethylsilane (TMS) (Sigma-Aldrich, St. Louis, MO, USA) as the internal standard.

### 2.3. Synthesis of 2-Chloro-N-((2-hydroxy-4-nitrophenyl)carbamothioyl)benzamide

**Figure 1:**
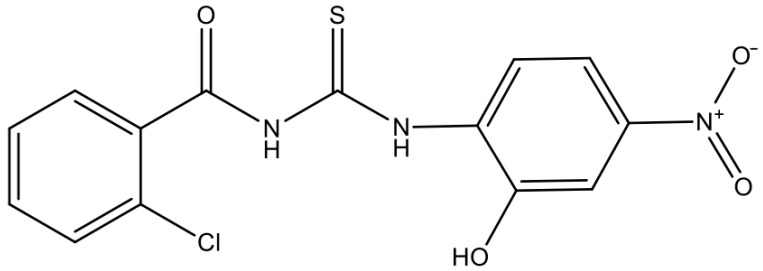
The molecular structure of HNCB.

Potassium thiocyanate (KSCN, 0.01 mol) was dissolved in 30 mL of acetone and added to a solution of 2-chlorobenzoyl chloride (0.01 mol) in 50 mL of acetone [50,51]. The mixture was refluxed for 30 min and then allowed to cool to room temperature. Subsequently, 2-amino-5-nitrophenol (0.01 mol), dissolved in 10 mL of acetone, was added, and the reaction mixture was stirred at room temperature for 2 h. After completion, the reaction mixture was poured into 300 mL of cold 0.1 M HCl, resulting in the formation of a precipitate. The solid was collected by filtration, washed with double-distilled water, and recrystallized using a mixture of ethanol and dichloromethane (C_2_H_5_OH/CH_2_Cl_2_). Figure 1 depicts the molecular structure of HNCB.

2-chloro-N-((2-hydroxy-4-nitrophenyl)carbamothioyl) benzamide (HNCB): White crystals, Yield 82%. FT-IR (KBr, cm^−1^): 3441–3360 (N-H), 3300 (O-H), 3070 (aromatic C-H), 1620 (C=O), 1560–1436 (aromatic C=C stretching vibrations, asymmetric NO_2_ stretching vibrations), 1373–1338 (Symmetric NO_2_ stretching vibrations), 1246–1226 (C-H in-plane bending), 1122 (C=S), 746 (C-Cl). ^1^H-NMR (400 MHz, DMSO-*d*_6_, ppm): 13.14 (1H-Phenolic O-H), 12.25 (1H, –NH–C=S), 11.58 (1H- –NH–C=O), 7.44–9.16 (7H, Aromatic-H). ^13^C-NMR (400 MHz, DMSO-*d*_6_, ppm): 177.9 (C=S), 168.3 (C=O), 109–148.9 (6C-Aromatic-C).

### 2.4. Fuel Properties and Blend Preparation

The fuel blends were precisely formulated in the laboratory using accurate volumetric measurements and thorough mixing to ensure homogeneity. Initially, the HNCB additive was dissolved in 10 mL of ethanol at three distinct concentrations (50, 100, and 200 ppm). These prepared solutions were subsequently introduced into 1000 mL of pure diesel fuel. All fuel blends were prepared immediately prior to testing and used within a short time frame to ensure homogeneity and to avoid potential phase separation during the experiments. Long-term storage stability and material compatibility assessments were beyond the scope of the present study and are suggested for future investigations. The full range of fuel mixtures, including the baseline pure diesel and the additive-enhanced blends at varying concentrations, is detailed in Table 1.

The key physicochemical properties of diesel and ethanol, such as density, lower heating value (LHV), viscosity, and other parameters relevant to engine performance and emission analysis, are presented in Table 2.

### 2.5. Engine Test Procedure

This section outlines the experimental procedures employed to assess the impact of HNCB, as a diesel fuel additive on engine performance and exhaust emissions. The experiments were carried out using a single-cylinder, air-cooled, four-stroke direct-injection diesel engine at the Automotive Technologies Application and Research Center of Sakarya University of Applied Sciences. Detailed specifications of the test engine, including the valve timing data and the injection parameters, are provided in Table 3.

**Figure 2:**
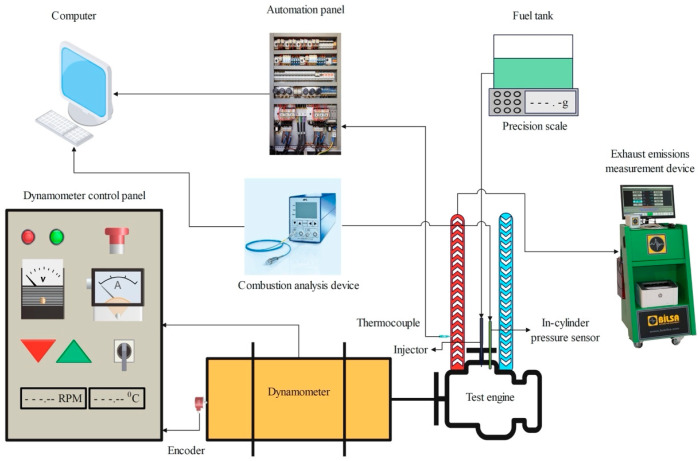
Schematic representation of the CI engine test bench and measurement system.

All experiments were conducted at a fixed engine speed of 1800 rpm, corresponding to the engine’s maximum torque point. The engine was tested under five distinct load conditions, representing torque values of 0 Nm, 6 Nm, 12 Nm, 18 Nm, and 24 Nm. For each load level, four EGR rates, 0%, 10%, 20%, and 30%, were applied to evaluate the effects systematically. An overview of the experimental setup and procedure is presented in Figure 2. In the present study, a cooled EGR system was employed. The recirculated exhaust gases were passed through a heat exchanger prior to being reintroduced into the intake manifold. This approach was selected to achieve effective NO_x_ mitigation while limiting excessive intake charge heating associated with hot EGR operation. Accordingly, the observed trends in combustion phasing, heat release characteristics, and emission behavior are interpreted within the context of cooled EGR operation.

Combustion analysis was performed using an AVL GH14P piezoelectric pressure sensor to measure in-cylinder pressure. The resulting pressure signals were amplified through an AVL charge amplifier and transmitted to the AVL Indi-Com combustion analysis system for processing. Crankshaft position was detected using an AVL crank angle encoder, which provided synchronized input to the Indi-Com system as part of the combustion diagnostics. CA5, CA10, CA50, and CA90 refer to the crank angle degrees corresponding to 5%, 10%, 50%, and 90% of the cumulative heat release, respectively. These parameters were used to assess combustion phasing, including the start of combustion, early combustion development, the location of the main heat release, and the late-stage completion tendency of the combustion event. The recorded in-cylinder pressure data were subjected to fourth-order filtering to eliminate noise prior to analysis. Fuel consumption was quantified by weighing the consumed fuel with a high-precision balance in conjunction with time measurements recorded by a chronometer. Fuel delivery was precisely regulated by an electronically controlled stepper motor integrated into the fuel pump assembly. Injection timing was not intentionally varied as an independent experimental parameter during the tests. The same injection system configuration was maintained for all fuel blends and operating conditions. EGR rate was likewise controlled via an electronically actuated valve system. Thermocouples were used to monitor both intake air and exhaust gas temperatures. Engine load was accurately adjusted using a Kemsan-Theory electric dynamometer control panel. All experimental variables, including EGR rate and throttle position, were monitored and logged in real time via a custom-developed control interface.

The uncertainty analysis was carried out in accordance with the methodologies prescribed by the Guide to the Expression of Uncertainty in Measurement (GUM). The overall combined standard uncertainty was evaluated by incorporating both Type A (statistical) and Type B (systematic) components. The analytical procedure for estimating uncertainty is presented in Equation (1) [57,58,59,60,61].

(1) ∆f=∂f∂x1Δx12+∂f∂x2Δx22+⋯∂f∂xnΔxn212

All uncertainty estimations were conducted based on the specifications of the measurement instruments, including resolution and manufacturer-stated accuracy limits. Additionally, repeated trials under stabilized operating conditions were analyzed to capture random variations and to assess the repeatability of the measurements. Standard uncertainty propagation techniques were applied in cases where multiple variables contributed to the final measurement results. The error bars shown in the relevant bar-chart figures represent the estimated measurement uncertainty range of the experimental system, based on instrument accuracy, measurement resolution, and uncertainty propagation. The uncertainties of the measuring instruments used in the experiments are listed in Table 4.

### 2.6. Emission Measurement and Calculation

The concentrations of NO_x_ and HC were measured in ppm (vol.) using an exhaust gas analyzer during the experiments. The exhaust gas concentrations measured by the gas analyzer were considered on a dry basis in the emission calculations. The measured concentrations were converted into specific emissions (g/kWh) assuming ideal gas behavior under standard temperature and pressure (STP) conditions. NO_x_ emissions were evaluated on an NO_2_-equivalent basis, while HC emissions were assessed on a propane-equivalent basis consistent with the reporting basis of the exhaust gas analyzer. Accordingly, molar masses of 46 g/mol for NO_2_ and 44 g/mol for HC were used in the ppm-to-mass conversions. The conversion from volumetric concentration (ppm) to mass concentration was performed using the following general expression:

(2) $$C_{i}\left(g/m^{3}\right)=\frac{{ppm}_{i}\;\times \;M_{i}\;}{22.414}$$

where *C_i_* is the mass concentration of the emission component (NO_x_ or HC), *ppm_i_* is the measured concentration in ppm, and *M_i_* is the corresponding molar mass (46 g/mol for NO_2_ and 44 g/mol for HC). Under EGR operation, the exhaust volumetric flow rate was estimated by scaling the reference (0-EGR) exhaust flow rate according to the applied EGR ratio:

(3) $${\dot{V}}_{exh,EGR}={\dot{V}}_{exh,0}\left(1-EGR\right)$$

The mass flow rate of each emission component was then calculated as:

(4) $${\dot{m}}_{i}\left(g/s\right)=C_{i}\;\times \;{\dot{V}}_{exh,EGR}$$

The effective engine power was determined from the measured torque values at a constant engine speed of 1800 rpm using:

(5) $$P_{e}\left(kW\right)=\frac{2\pi NT}{60,000}$$

where *N* is the engine speed (rpm) and *T* is the engine torque (Nm).

Finally, the specific emissions of NO_x_ and HC were obtained by normalizing the corresponding mass flow rates with the effective engine power:

(6) $${Emission}_{i}\left(g/kWh\right)=\frac{{\dot{m}}_{i}\;\times \;3600}{P_{e}}$$

The emission conversion and power calculation procedures were performed in accordance with standard engine testing methodologies reported in the literature and relevant international standards [62,63]. At zero-load operating conditions (0 Nm), the effective engine power is zero; therefore, specific emissions were not reported for this condition.

## 3. Results and Discussion

In this study, a novel compound belonging to the class of aroylthiourea derivatives containing nitrogen and sulfur, namely 2-chloro-N-((2-hydroxy-4-nitrophenyl)carbamothioyl)benzamide (HNCB), was synthesized for the first time. Its structural characterization was thoroughly performed using FT-IR, 1H-NMR, and 13C-NMR spectroscopic techniques. Following the successful synthesis and characterization, HNCB was evaluated as a fuel additive to investigate its impact on engine performance and emission characteristics. Specifically, the effects of HNCB and ethanol additives were examined in a single-cylinder diesel engine under varying engine loads and exhaust gas recirculation (EGR) rates. The results obtained provide insights into the potential of HNCB as an alternative fuel additive for improving combustion efficiency and reducing harmful emissions.

### 3.1. FT-IR Studies

**Figure 3:**
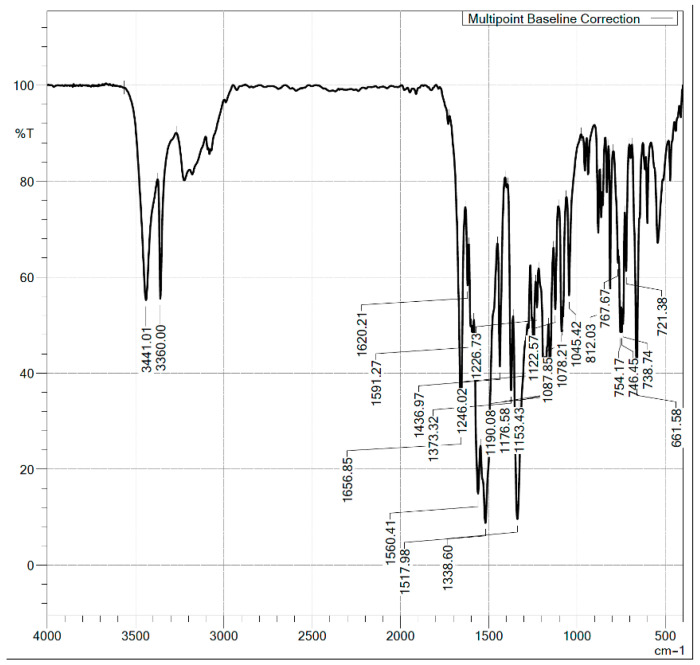
The FT-IR spectrum of HNCB.

The FT-IR spectrum of 2-chloro-N-((2-hydroxy-4-nitrophenyl)carbamothioyl)benzamide (HNCB) is depicted in Figure 3. The doublet peaks appearing within the 3441–3360 cm^−1^ range are assigned to the symmetric stretching vibrations of the amino (–NH2) functional group. The broad absorption band observed at 3300 cm^−1^ is attributed to the stretching vibration of the phenolic –OH group. The aromatic C–H stretching vibrations appear at 3070 cm^−1^. The strong absorption band observed at 1620 cm^−1^ is attributed to the C=O stretching vibrations, indicating the presence of a carbonyl group. The bands observed at 1122 cm^−1^ are assigned to the C=S stretching vibrations. The symmetric stretching modes of the NO_2_ group appear within the 1373–1338 cm^−1^ range, whereas the asymmetric stretching vibrations are identified in the 1560–1436 cm^−1^ region [64]. Additionally, overlapping absorption bands corresponding to aromatic C=C stretching vibrations are also present in this range. The in-plane bending vibrations of the C–H bonds are indicated by bands in the 1246–1226 cm^−1^ range. Additionally, the C–Cl stretching vibration is identified at 746 cm^−1^. These spectral features confirm the successful synthesis of 2-chloro-N-((2-hydroxy-4-nitrophenyl)carbamothioyl)benzamide and the presence of its characteristic functional groups.

### 3.2. NMR Spectral Analysis

**Figure 4:**
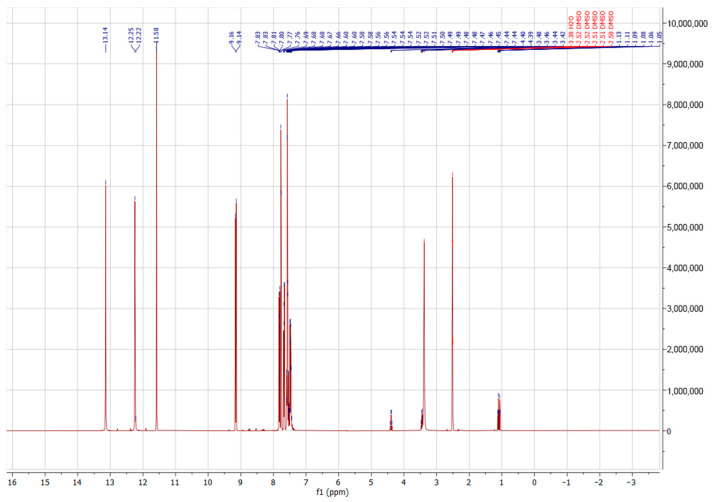
The ^1^H-NMR spectrum of 2-chloro-N-((2-hydroxy-4-nitrophenyl)carbamothioyl)benzamide.

The ^1^H-NMR spectrum of 2-chloro-N-((2-hydroxy-4-nitrophenyl) carbamothioyl) benzamide is given in Figure 4. In the spectrum, a singlet observed at 13.14 ppm was assigned to the phenolic –OH proton (1H, Phenolic-H). The high chemical shift of this signal is attributed to intramolecular hydrogen bonding with the adjacent nitro group, which causes deshielding of the hydroxyl proton. Two amide-type N-H protons corresponding to the thiourea moiety were detected between 11.58 ppm and 12.25 ppm. These signals are characteristic of the –NH–C=S and –NH–C=O functional groups. The NH proton attached to the C=S group is slightly more downfield due to the lower electronegativity of sulfur compared with oxygen, which allows for stronger deshielding. The aromatic region of the spectrum exhibited multiple signals in the range of 7.44–9.16 ppm corresponding to the seven aromatic protons present in the molecule.

**Figure 5:**
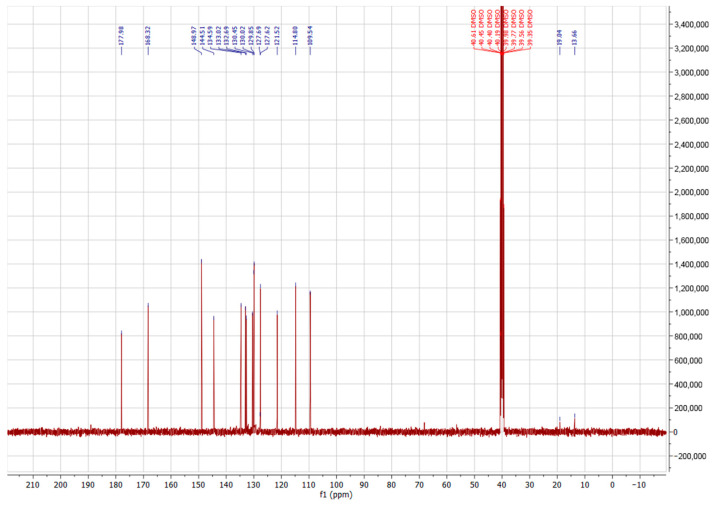
The ^13^C-NMR spectrum of 2-chloro-N-((2-hydroxy-4-nitrophenyl)carbamothioyl)benzamide.

^13^C-NMR spectrum of 2-chloro-((2-hydroxy-4-nitrophenyl)carbamothioyl)-2-chlorobenzamide is given in Figure 5. In the ^13^C-NMR spectrum of the synthesized compound, several distinct signals were observed that support the proposed structure. The resonances between 109.5 and 148.9 ppm correspond to the sp^2^-hybridized carbon atoms of the aromatic rings, confirming the presence of phenyl groups within the molecular framework. These chemical shifts are in accordance with typical values reported for aromatic carbon atoms. A notable peak observed at 168.3 ppm is attributed to the carbonyl (C=O) carbon, which is deshielded due to the electronegative oxygen and the double bond character. This chemical shift is consistent with those reported in the literature for similar amide derivatives, further supporting the presence of the amide moiety in the structure. Additionally, the peak observed at 177.9 ppm is assigned to the thiocarbonyl (C=S) carbon. The downfield shift of this carbon is characteristic of C=S bonds, which tend to appear at higher chemical shift values due to the lower electronegativity of sulfur and the reduced electron shielding compared with oxygen. This signal confirms the successful incorporation of the thiourea functionality in the synthesized compound. The overall ^13^C-NMR spectrum is in good agreement with previously published data for structurally related thiourea and benzoylthiourea compounds providing strong evidence for the structural integrity of the synthesized molecule [65,66].

### 3.3. Experimental Results

**Figure 6:**
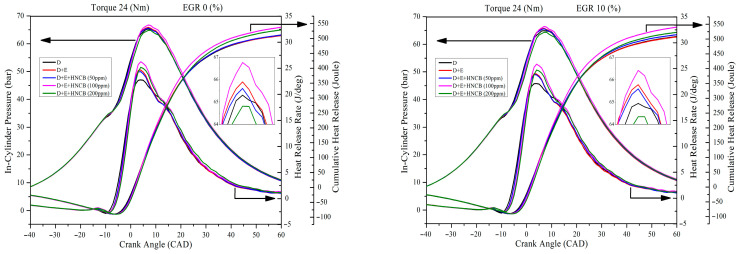

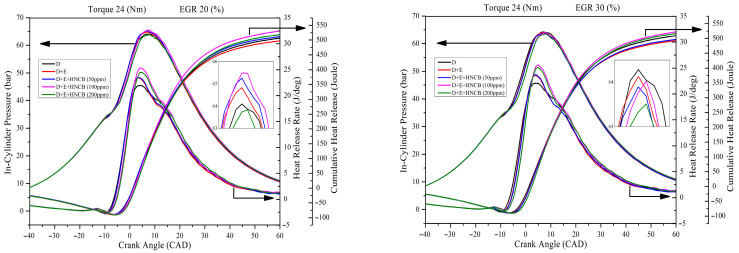
The effects of EGR and ethanol/HNCB additive to diesel fuel on cylinder pressure, CHR and HRR at 24 Nm torque.

The effects of various fuel blends and EGR rates on combustion characteristics, specifically cylinder pressure, cumulative heat release (CHR), and heat release rate (HRR), at a constant engine torque of 24 Nm, as presented in Figure 6. The cylinder pressure curves show that pure diesel (D) produced the highest peak pressure under no EGR conditions. As the EGR rate increased, a consistent decline in peak pressure was observed across all fuel types. This can be attributed to the reduced oxygen concentration in the intake mixture due to the recirculated exhaust gases, which lowers the combustion temperature and slows the flame propagation rate. Interestingly, blends containing ethanol and HNCB additive maintained higher peak pressures than pure diesel under the same EGR conditions. This improvement is likely due to ethanol’s inherent oxygen content and better volatility, which enhance fuel–air mixing and combustion intensity. Particularly at higher additive concentrations (100 ppm and 200 ppm), the cylinder pressure profiles indicate a more efficient combustion process, even with increased EGR. In terms of cumulative heat release (CHR), a general reduction and delay in the heat release profile were observed with increasing EGR. This outcome is consistent with the reduction in combustion temperature and prolonged ignition delay under EGR conditions. However, the ethanol/HNCB blends mitigated these effects, exhibiting higher CHR values, especially at 100 ppm and 200 ppm additive levels. It is also noteworthy that the increase in HNCB concentration from 100 ppm to 200 ppm did not further enhance cylinder pressure or HRR. Instead, the 200 ppm blend showed a reduction in both parameters compared with the 100 ppm blend, indicating a non-linear additive concentration effect. This decrease may be related to excessive additive dosing, which can adversely affect mixture homogeneity, local oxidation, and combustion development under EGR-diluted conditions. Thus, 100 ppm HNCB appears to be closer to the optimum concentration for maintaining stronger pressure development and heat release behavior at 24 Nm. This enhancement suggests that the presence of oxygenated and volatile fuel components compensates for the oxygen-deficient environment by promoting improved pre-mixture formation and a more complete combustion process. The heat release rate (HRR) curves further support these findings. With higher EGR rates, a noticeable decline in the peak HRR was recorded, indicating slower and less intense combustion. Yet, fuels blended with ethanol and HNCB additives displayed sharper and higher HRR peaks than diesel alone. These sharper peaks can be linked to the superior atomization and ignition properties of ethanol, as well as the catalytic effects of HNCB on flame propagation. The increased HRR in these cases implies a more vigorous and efficient combustion event. These trends align with findings from prior studies that oxygenated fuels such as ethanol can enhance combustion performance and mitigate the adverse impacts of EGR under light and medium loads [67,68]. However, they also noted that excessive EGR could lead to incomplete combustion and increased emissions of unburned hydrocarbons and CO due to poor oxygen availability. In conclusion, the results demonstrate that ethanol and HNCB additives have a beneficial effect on diesel combustion under EGR conditions. By improving cylinder pressure, CHR, and HRR profiles, these additives help maintain efficient combustion even under oxygen-limited environments. Nonetheless, careful optimization of additive concentration and EGR rate is essential to avoid combustion instability at higher dilution levels.

The observed enhancement in cylinder pressure and heat release characteristics in the presence of HNCB can be attributed to its heteroatom-rich molecular structure. The thiourea moiety (–NH–C=S–NH–) is thermally labile under combustion conditions and can undergo homolytic cleavage, generating reactive radical intermediates that may participate in chain-branching reactions. These radicals can accelerate the early stages of combustion, particularly in the premixed phase, leading to higher peak pressures and sharper HRR profiles. Additionally, the nitro (–NO) substituent present in the aromatic ring may act as an internal oxygen donor under high-temperature conditions, facilitating localized oxidation reactions even under oxygen-deficient environments such as high EGR operation. Furthermore, the aromatic backbone enhances fuel miscibility and promotes more uniform dispersion of the additive within the diesel–ethanol matrix. The combined effect of improved radical chemistry and enhanced local oxidation kinetics explains the observed increase in CHR and HRR, especially at the optimal 100 ppm concentration.

**Figure 7:**
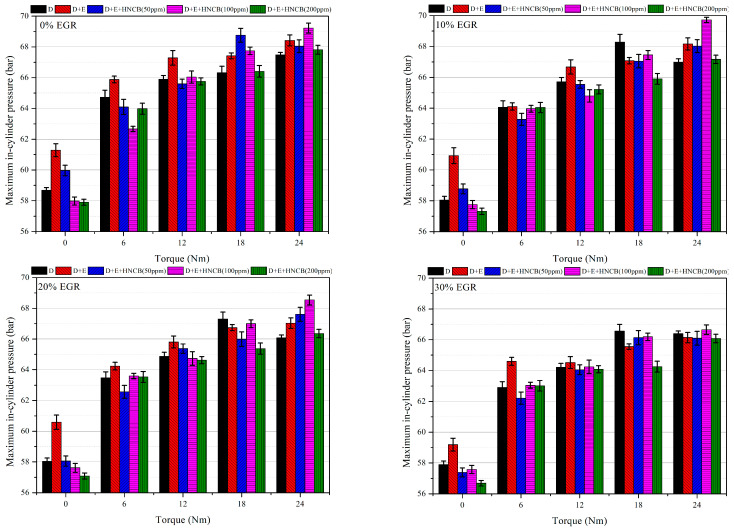
The effects of EGR and ethanol/HNCB additive to diesel fuel on maximum in-cylinder pressure. Error bars indicate the estimated measurement uncertainty range of the experimental system.

Figure 7 illustrates how different fuel blends and EGR rates influence maximum in-cylinder pressure across varying engine loads. The results indicate that both EGR and fuel composition play critical roles in shaping combustion intensity. Across all EGR conditions, increasing torque results in higher peak pressures due to elevated fuel demand. At 0% EGR, the D + E + HNCB (100 ppm) blend produces the highest pressure (70.22 bar) at 24 Nm, outperforming pure diesel (67.48 bar). The increase in maximum in-cylinder pressure observed for the HNCB-containing blends, particularly at 100 ppm, can be linked to improved premixed combustion intensity. The presence of both nitrogen and sulfur atoms in the HNCB structure facilitates intermediate radical formation during thermal decomposition, which enhances ignition reactivity. At optimal concentrations, this leads to more synchronized ignition and rapid energy release in the early combustion phase. However, at higher concentrations (200 ppm), excessive additive loading may alter local equivalence ratios and increase mixture heterogeneity, resulting in delayed combustion and reduced pressure development, especially under low-load conditions. Together, these effects promote more effective energy release during the premixed combustion phase. As EGR increases to 10%, 20%, and 30%, a general decline in peak pressure is observed for all fuels. This is primarily due to the reduced oxygen availability and lower combustion temperatures caused by the recirculated exhaust gases. However, the D + E + HNCB (100 ppm) blend consistently maintains higher or comparable pressures than diesel. For instance, at 30% EGR and 24 Nm, it achieves 66.65 bar, slightly surpassing diesel at 66.39 bar, indicating that the combined oxygenated fuel formulation helps mitigate EGR-induced combustion weakening under high-load conditions. In contrast, low-load (0–6 Nm) conditions show less pronounced benefits. In some cases, especially with 200 ppm HNCB, the peak pressures drop below those of diesel. This may result from incomplete vaporization and lower combustion temperatures, which reduce the effectiveness of both ethanol and the additive under these conditions. At medium loads (12–18 Nm), HNCB-enhanced blends regain their advantage, particularly at 100 ppm concentration. The data suggest that proper additive dosing is critical; excessive HNCB may destabilize the mixture or delay ignition, while optimal levels can significantly enhance combustion quality under diluted intake conditions. Overall, the study shows that D + E + HNCB (100 ppm) offers the most balanced performance, especially under high torque and moderate EGR conditions. These findings are consistent with previous studies reporting that oxygenated additives can effectively mitigate EGR-induced combustion deterioration and help maintain engine efficiency [69,70].

**Figure 8:**
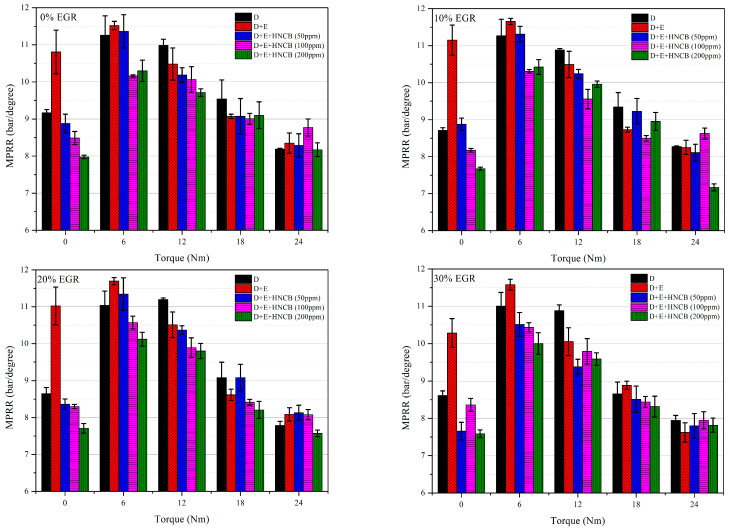
The effects of EGR and ethanol/HNCB additive to diesel fuel on MPRR. Error bars indicate the estimated measurement uncertainty range of the experimental system.

Figure 8 and the accompanying data provide insight into the impact of EGR and ethanol/HNCB additives on the maximum pressure rise rate (MPRR), which is a critical parameter in evaluating the rapidity and stability of the combustion process. MPRR reflects the rate of pressure increase during the premixed combustion phase and is directly associated with engine noise, mechanical stress, and combustion quality. At 0% EGR, pure diesel and D + E blends exhibit the highest MPRR values at low and medium torque levels. The D + E blend reaches 12.32 bar/ms at 6 Nm, indicating a highly aggressive combustion onset due to ethanol’s volatility and oxygen content. However, the inclusion of HNCB at 100 ppm moderates the pressure rise rate (10.16 bar/ms), suggesting a more controlled combustion. At high torque (24 Nm), MPRR decreases across all fuels, with D + E + HNCB (100 ppm) again providing a balance between performance and smooth pressure development. The moderation of MPRR values with HNCB addition suggests a dual role of the additive in combustion kinetics. While the thiourea-derived radicals promote ignition, the relatively complex aromatic structure of HNCB may slow down flame propagation slightly, leading to a more controlled pressure rise. This balance is particularly evident at 100 ppm, where combustion remains sufficiently rapid for efficiency while avoiding excessively sharp pressure gradients. In contrast, at 200 ppm, the increased concentration may suppress flame propagation due to localized quenching effects or diffusion limitations, resulting in reduced MPRR values.

As EGR is introduced, a general decline in MPRR is observed, driven by reduced oxygen levels and flame temperature. For example, at 30% EGR and 6 Nm, diesel records an MPRR of 11.00 bar/ms, while D + E + HNCB (100 ppm) yields 10.43 bar/ms, still preserving combustion efficiency. Notably, HNCB at 100 ppm consistently maintains MPRR values close to diesel, even under high EGR rates, highlighting its potential to stabilize combustion under diluted conditions. Conversely, the 200 ppm HNCB additive leads to a significant reduction in MPRR across all torques and EGR levels. At 24 Nm and 10% EGR, the MPRR drops to just 7.16 bar/ms, which may indicate delayed ignition or incomplete premixed combustion, likely caused by over-dilution or additive saturation. These findings imply that while HNCB enhances combustion control at moderate concentrations, excessive use can diminish the pressure development rate and potentially impair combustion. In conclusion, ethanol-based HNCB additives, especially at 100 ppm concentration, offer promising improvements in controlling pressure rise rates, balancing efficiency, and engine durability. However, optimal dosing is critical; overly high additive levels may compromise combustion sharpness and increase cycle-to-cycle variability, particularly under high EGR and low load conditions [71,72].

**Figure 9:**
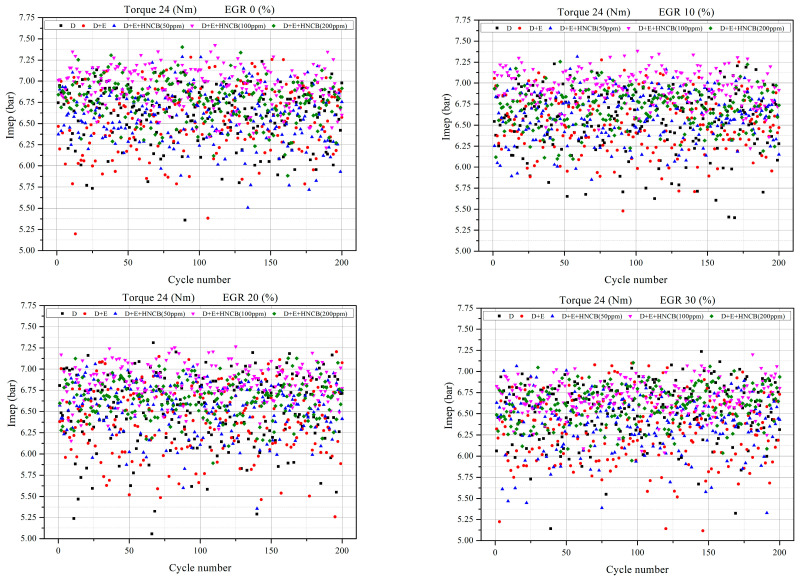
The effects of EGR and ethanol/HNCB additive to diesel fuel on IMEP cyclic variation.

Figure 9 illustrates the cyclic variation in Indicated Mean Effective Pressure (IMEP) under various EGR levels and fuel compositions. IMEP cyclic variation is a crucial indicator of combustion stability, with higher fluctuations suggesting unstable combustion, which can adversely affect performance, emissions, and engine durability. At 0% EGR, pure diesel (D) exhibits the widest IMEP distribution, indicating higher cycle-to-cycle variability and less consistent combustion. The addition of ethanol (D + E) significantly reduces the scatter of IMEP values, attributed to ethanol’s improved vaporization and its oxygen content, which enhance the mixture homogeneity and combustion repeatability. However, the most notable reduction in IMEP variability is observed with the D + E + HNCB (100 ppm) blend. This fuel formulation maintains a tight cluster of IMEP values, reflecting more uniform and repeatable combustion events. The HNCB additive likely influences ignition-related combustion behavior and flame development characteristics, as reflected by changes in IMEP cyclic variation and early combustion stability under different EGR conditions. As the EGR rate increases to 10%, 20%, and 30%, a general increase in IMEP cyclic variation is observed across all fuel types, which is expected due to the lower oxygen availability and cooler combustion temperatures associated with exhaust gas recirculation. Nevertheless, D + E + HNCB (100 ppm) continues to demonstrate better cyclic stability compared with other blends, even under 30% EGR. Its consistent performance indicates that this blend helps mitigate the destabilizing effects of EGR by promoting a more reliable combustion process. On the other hand, D + E + HNCB (200 ppm) shows a slight increase in IMEP dispersion at higher EGR rates, particularly noticeable at 30% EGR. This suggests that excessive additive concentration may lead to over-retarded ignition or suboptimal mixture formation, reducing the benefits of enhanced oxygenation and potentially leading to local misfires or incomplete combustion. In summary, the addition of ethanol and moderate HNCB concentrations effectively improves combustion stability, as evidenced by reduced IMEP cyclic variation. Among the tested configurations, D + E + HNCB at 100 ppm offers the most favorable balance, delivering superior cycle-to-cycle consistency and resilience against EGR-induced instability. The reduction in IMEP cyclic variability with HNCB addition can be explained by improved ignition consistency and more homogeneous combustion conditions. The polar functional groups (–NO_2_, –OH, –NH–) in HNCB may enhance intermolecular interactions within the fuel blend, promoting better atomization and vapor-phase mixing. Additionally, the presence of heteroatoms facilitates more predictable radical formation pathways during ignition, reducing cycle-to-cycle variability. However, excessive additive concentration (200 ppm) may lead to local mixture stratification and delayed ignition, thereby increasing combustion instability. These observed improvements in early combustion stability and pressure development can be attributed to the physicochemical characteristics of the HNCB additive. The nitrogen- and sulfur-containing functional groups in the HNCB molecule may influence combustion through complementary physicochemical pathways. Nitrogen-bearing moieties can facilitate early-stage oxidation and radical formation during the premixed combustion phase, contributing to improved ignition stability. Sulfur-containing structures may affect local reaction kinetics and heat release behavior by influencing fuel decomposition processes. In addition, the polar nature of these functional groups can enhance fuel–air interaction, particularly under diluted intake conditions such as high EGR operation. Together, these effects offer a plausible explanation for the observed improvements in combustion stability and early heat release characteristics without implying direct catalytic activity. These findings support previous literature emphasizing the importance of fuel-bound oxygen and ignition-enhancing additives in promoting stable and efficient diesel engine combustion, and reducing cyclic variability under dilute operating conditions [73].

**Figure 10:**
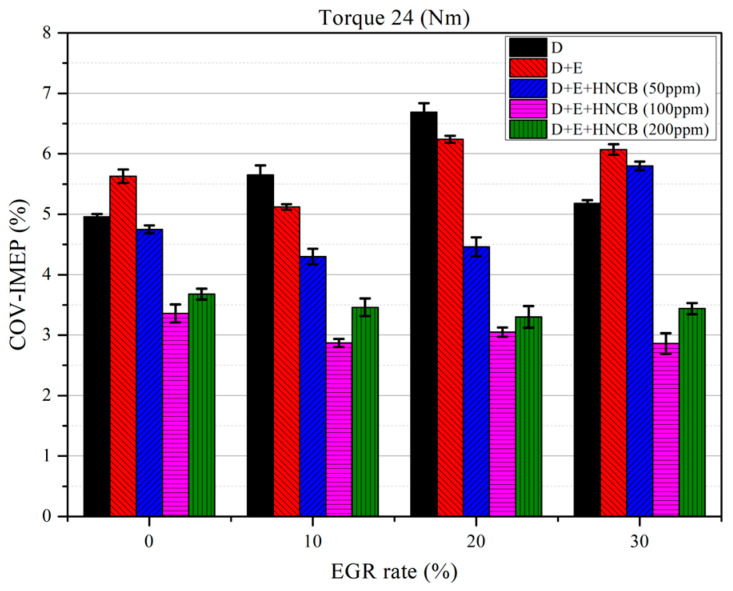
Variation in COV_IMEP at 24 Nm engine load under different EGR rates for diesel fuel with ethanol and HNCB additive (50–200 ppm). Error bars indicate the estimated measurement uncertainty range of the experimental system.

In Figure 10, the variation in COV_IMEP with EGR ratio shows that combustion stability depends strongly on both EGR dilution and fuel composition. Since COV_IMEP values below approximately 5% are generally regarded as acceptable for stable engine operation, the values closer to 3% observed for D + E + HNCB (100 ppm) indicate more repeatable combustion behavior. The diesel–ethanol blend showed a fluctuating effect on cycle-to-cycle variations compared with neat diesel; therefore, ethanol addition alone did not provide a consistent improvement in combustion stability across all EGR rates.

The variation in COV_IMEP with EGR ratio demonstrates that combustion stability is strongly affected by both exhaust gas recirculation and fuel composition. The diesel–ethanol blend showed a fluctuating effect on cycle-to-cycle variations compared with neat diesel. Although D + E exhibited lower COV_IMEP values than diesel at some EGR ratios, its cyclic variability increased noticeably at 0% and 30% EGR. This behavior may be related to the lower cetane number and higher latent heat of vaporization of ethanol, which can prolong ignition delay and locally reduce in-cylinder temperature, particularly under highly diluted combustion conditions. Therefore, ethanol addition alone did not provide a consistent improvement in combustion stability across all EGR rates. In contrast, the incorporation of HNCB into the D + E blend significantly improved combustion stability depending on the additive concentration. Among all tested fuel blends, D + E + HNCB (100 ppm) consistently showed the lowest COV_IMEP values, ranging between approximately 2.86% and 3.36%. This indicates that the 100 ppm HNCB concentration provided the most favorable effect on cycle-to-cycle combustion repeatability. The improvement may be associated with enhanced combustion phasing, more stable heat release behavior, and improved ignition-related characteristics under EGR-diluted conditions. The 200 ppm HNCB blend also reduced COV_IMEP compared with D and D + E, although its effect was slightly weaker than that of the 100 ppm concentration. This suggests that increasing the additive concentration beyond an optimum level does not necessarily provide further improvement in combustion stability. The 50 ppm HNCB blend showed an intermediate and less consistent behavior. While it reduced COV_IMEP compared with D + E at several EGR conditions, its COV_IMEP value increased noticeably at 30% EGR. This indicates that the 50 ppm concentration may be insufficient to maintain the same level of combustion stability under highly diluted conditions. Overall, the results confirm that HNCB addition, particularly at 100 ppm, effectively improves combustion stability and compensates for the instability tendency associated with ethanol blending and EGR dilution.

**Figure 11:**
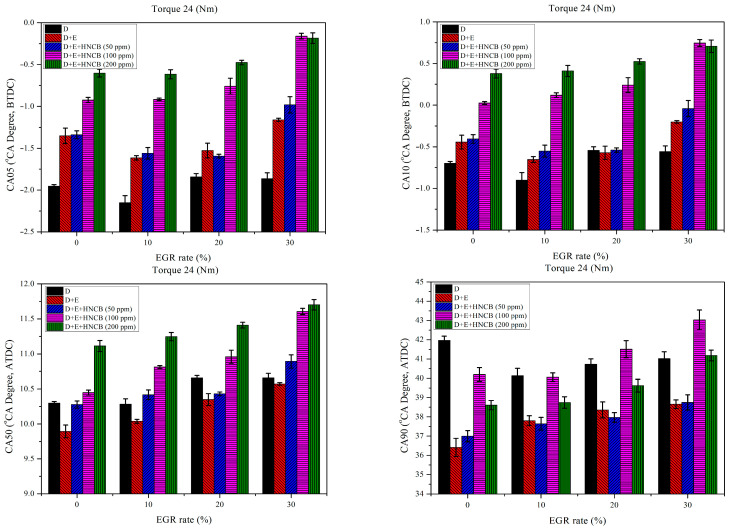
The effects of EGR and ethanol/HNCB additive to diesel fuel on CA5, CA10, CA50 and CA90. Error bars indicate the estimated measurement uncertainty range of the experimental system.

Figure 11 and the corresponding data summarize the effects of EGR rate and ethanol/HNCB fuel blends on the combustion phasing parameters CA5, CA10, CA50, and CA90, which represent the crank angle locations corresponding to 5%, 10%, 50%, and 90% of the cumulative heat release, respectively. These parameters are essential for assessing combustion delay, ignition timing, combustion duration, and completeness. Since injection timing was not intentionally varied in this study, the observed CA05 variations were mainly interpreted in relation to fuel formulation, EGR dilution, and load-dependent changes in fuel delivery rather than an imposed injection timing strategy. At CA05, which indicates the start of combustion, a clear trend of retardation is observed with increasing EGR across all fuel types. Pure diesel exhibits the earliest CA05 timings (e.g., −2.15 °CA at 10% EGR), while D + E + HNCB (200 ppm) shows the most delayed ignition (−0.60 °CA at 0% EGR). This delay is expected with EGR due to reduced oxygen and lower in-cylinder temperatures. However, the presence of ethanol and HNCB mitigates this effect to some extent. Notably, HNCB at 100 ppm provides the best balance, with a moderate ignition delay (e.g., −0.92 °CA at 0% EGR), suggesting an optimal condition for controlled premixed combustion. CA10, the early combustion phase marker, follows a similar pattern. At 30% EGR, diesel reaches −0.56 °CA, while D + E + HNCB (200 ppm) advances into positive timing (+0.70 °CA), indicating significantly delayed ignition. In contrast, D + E + HNCB (100 ppm) exhibits ideal CA10 timing, remaining slightly before TDC across all EGR levels, which helps optimize combustion phasing and reduce pressure rise rate irregularities. For CA50, representing the point of maximum energy release, advanced timings are desired for efficiency. Here, ethanol and HNCB blends outperform diesel. At 30% EGR, diesel peaks at 10.65 °CA, while D + E + HNCB (200 ppm) reaches 11.70 °CA. The addition of HNCB leads to earlier energy release, improving thermal efficiency. Lastly, CA90, indicating the near completion of combustion, highlights the duration of the burn. Ethanol and HNCB blends significantly shorten combustion duration. While diesel completes combustion around 41.0 °CA, D + E + HNCB (100 ppm) finishes near 43.0 °CA, suggesting a more extended and efficient combustion tail, improving emissions and completeness of oxidation. In conclusion, ethanol and HNCB additives substantially affect combustion phasing. D + E + HNCB at 100 ppm offers the best trade-off across CA05 to CA90, enabling controlled ignition, efficient energy release, and complete combustion, even under high EGR rates.

The influence of HNCB on combustion phasing can be attributed to its effect on ignition delay and heat release progression. The nitrogen-containing functional groups can facilitate early radical formation, thereby slightly advancing ignition timing at moderate concentrations. Simultaneously, the aromatic and thiocarbonyl (C=S) components may slow down the later stages of combustion, leading to a broader heat release profile. This dual behavior results in optimized CA50 positioning, which is critical for maximizing thermal efficiency. At higher concentrations, however, the dominance of diffusion-controlled combustion leads to delayed CA values and extended combustion duration.

**Figure 12:**
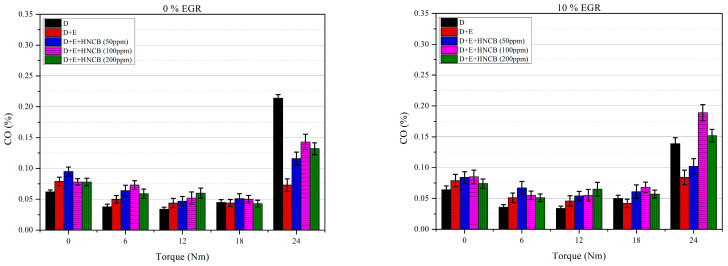

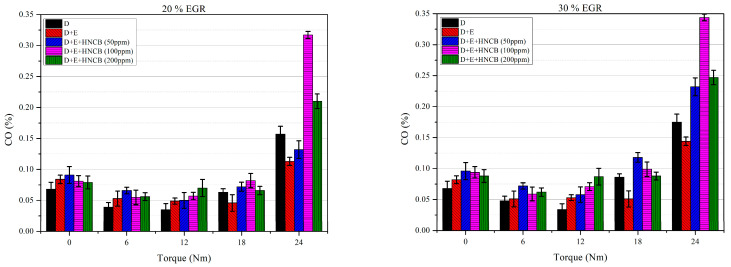
The effects of EGR and ethanol/HNCB additive to diesel fuel on CO emissions. Error bars indicate the estimated measurement uncertainty range of the experimental system.

Figure 12 illustrates the impact of varying EGR rates and ethanol/HNCB fuel blends on carbon monoxide (CO) emissions across different engine loads. CO emissions are strongly influenced by local equivalence ratios, combustion temperature, and oxygen availability. Hence, both exhaust gas recirculation and fuel formulation play significant roles. At 0% EGR, CO emissions remain relatively low across all fuel types at partial loads. Diesel fuel exhibits the lowest emissions (0.034–0.062), while blends containing ethanol and HNCB, especially at 100 ppm, show a modest increase. This increase is due to the oxygenated nature of ethanol, which promotes leaner local mixtures that can inhibit full oxidation at low combustion temperatures. However, at 24 Nm torque, the D + E + HNCB (100 ppm) blend records a significant CO emission level (0.143), compared with diesel (0.214), indicating improved combustion efficiency under high-load conditions. As EGR increases, a progressive rise in CO emissions is observed, particularly at high torque. This is expected due to the dilution of intake air with inert gases, which lowers in-cylinder temperatures and oxygen concentration, hindering complete oxidation of fuel. Nevertheless, ethanol and HNCB additives demonstrate a partial compensatory effect. For instance, at 30% EGR and 24 Nm, diesel emits 0.175 g/kWh CO, whereas D + E + HNCB (100 ppm) reaches 0.344 g/kWh. Although higher in absolute terms, this is accompanied by higher combustion efficiency and smoother heat release, as previously discussed. Among the blends, D + E + HNCB (100 ppm) consistently produces the highest CO emissions under high load and high EGR, which may be attributed to the rich premixed zones formed by rapid vaporization and inadequate time for oxidation in cooler environments. In contrast, D + E + HNCB (50 ppm) offers a more balanced performance, with moderate CO levels and reduced sensitivity to EGR rates. Interestingly, at low and mid torques, the HNCB additives reduce CO emissions, particularly when compared with the D + E blend alone. This suggests that HNCB helps stabilize combustion and ensures better oxidation in early flame propagation stages. However, at high loads and high EGR, its influence becomes limited by physical conditions, such as low oxygen and temperature thresholds. Ethanol and HNCB additives affect CO emissions differently depending on torque and EGR level. The variation in CO emissions with HNCB addition reflects the balance between improved combustion initiation and limitations in oxidation under oxygen-deficient conditions. At low and moderate additive concentrations, the enhanced radical formation promotes more complete oxidation of carbon species, reducing CO emissions. However, under high EGR and high load conditions, the limited oxygen availability combined with rapid heat release may restrict the complete oxidation of CO to CO_2_. Additionally, excessive HNCB (200 ppm) may lead to locally rich zones due to altered spray characteristics, further contributing to incomplete combustion and elevated CO levels. While they promote better combustion at partial load, careful additive optimization is required to prevent excessive CO formation under heavily diluted conditions [74,75,76].

**Figure 13:**
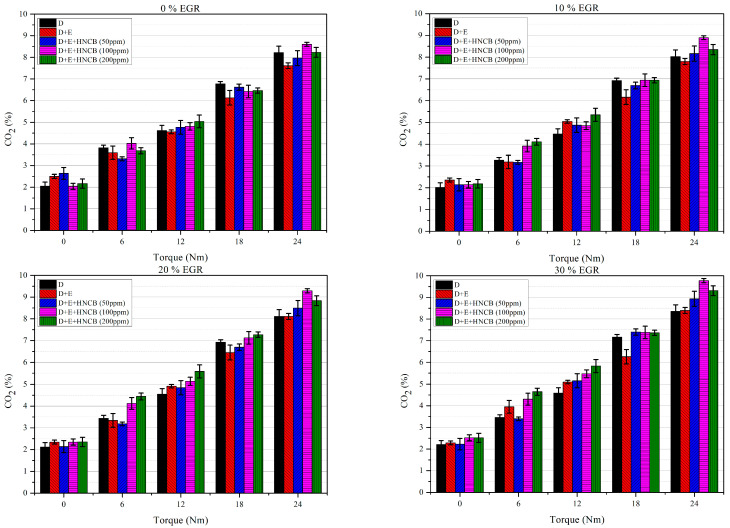
The effects of EGR and ethanol/HNCB additive to diesel fuel on CO_2_ emissions. Error bars indicate the estimated measurement uncertainty range of the experimental system.

Figure 13 presents the variation in CO_2_ emissions as a function of engine load, EGR rate, and fuel composition. CO_2_ is a direct indicator of complete combustion, as it reflects the degree to which carbon in the fuel is oxidized. Therefore, higher CO_2_ emissions are generally associated with improved combustion efficiency. At 0% EGR, pure diesel and D + E fuels yield moderate CO_2_ values, which increase with rising engine load due to higher fuel consumption. For instance, at 24 Nm, diesel emits 8.22%, while D + E emits 7.61%. Notably, the D + E + HNCB (100 ppm) blend records a higher CO_2_ level (8.60%) at the same load, indicating more effective carbon oxidation due to improved atomization, faster ignition, and better mixing. This trend is consistent across mid-to-high loads, demonstrating the positive influence of HNCB at 100 ppm in enhancing combustion quality. As EGR is introduced (10–30%), CO_2_ emissions generally increase with torque but show a strong dependency on fuel formulation. At 30% EGR and 24 Nm, CO_2_ emissions peak for D + E + HNCB (100 ppm) at 9.77%, followed closely by the 200 ppm variant (9.31%). These elevated values, despite high EGR dilution, suggest that ethanol and HNCB maintain efficient combustion by compensating for the oxygen-deficient environment. In contrast, pure diesel shows slightly lower CO_2_ emissions (8.35%), which may reflect incomplete combustion under the same conditions. Interestingly, at low loads (0–6 Nm), CO_2_ emissions remain relatively low across all fuel types, but blends with HNCB tend to exhibit slightly higher values than diesel or D + E. This supports the notion that oxygenated additives enable more complete combustion even when the engine operates under conditions with poor mixing and low in-cylinder temperature. Additionally, the increasing CO_2_ trend with both load and additive concentration implies that ethanol and HNCB not only enhance energy conversion efficiency but also help mitigate the adverse effects of EGR on oxidation. However, at very high additive levels (200 ppm), the improvement plateaus, and in some cases, CO_2_ levels are slightly reduced compared with the 100 ppm variant, suggesting that overdosage may not provide further benefit. D + E + HNCB at 100 ppm emerges as the most effective combination in maximizing CO_2_ output, and by extension, combustion completeness, even under elevated EGR conditions. The increase in CO_2_ emissions observed with HNCB addition, particularly at 100 ppm, indicates improved combustion completeness. The presence of oxygen-containing functional groups and the enhanced radical pool facilitate the oxidation of intermediate species such as CO and unburned hydrocarbons. Furthermore, the improved atomization and mixing characteristics associated with the additive contribute to a more homogeneous combustion process, resulting in higher carbon conversion efficiency.

**Figure 14:**
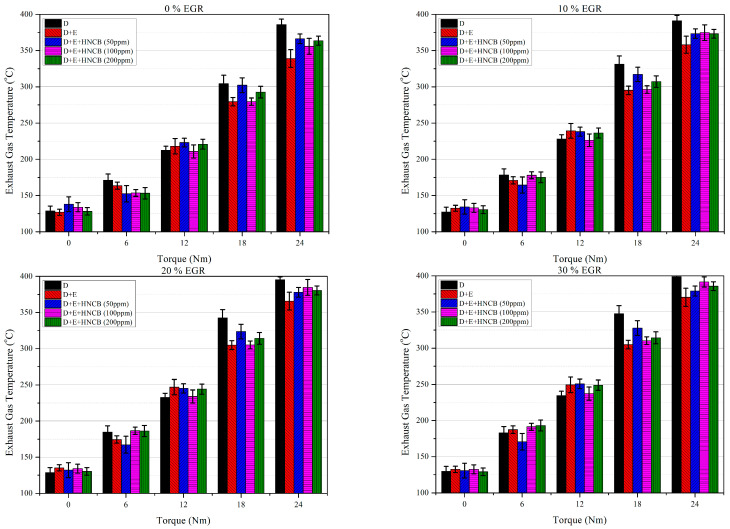
The effects of EGR and ethanol/HNCB additive to diesel fuel on EGT. Error bars indicate the estimated measurement uncertainty range of the experimental system.

Figure 14 demonstrates the variation in Exhaust Gas Temperature (EGT) with engine torque for different fuel compositions and EGR levels. EGT is a critical parameter that reflects in-cylinder combustion intensity and thermal energy transfer efficiency. Higher EGTs generally indicate more intense combustion and can influence engine durability and emission control strategies.

At 0% EGR, diesel fuel exhibits a gradually increasing EGT with torque, peaking at 385.85 °C at 24 Nm. The D + E blend shows slightly lower values at low-to-mid loads, but a significant drop (339.10 °C) at high loads. Interestingly, the addition of HNCB enhances EGT, particularly at 50 ppm and 100 ppm, reaching 366.26 °C and 355.94 °C, respectively. These elevated temperatures suggest improved combustion efficiency due to the oxygenated nature of ethanol and the combustion-promoting characteristics of HNCB. However, at low torque (e.g., 0 Nm), HNCB (100 ppm) provides a higher EGT (133.89 °C) compared with diesel (128.60 °C), indicating quicker heat release due to improved ignition. As EGR increases, overall EGT values decrease slightly at low loads due to reduced oxygen concentration and lower peak temperatures, yet the effect is nuanced at higher loads. At 30% EGR, diesel yields 398.80 °C at 24 Nm, while D + E + HNCB (100 ppm) reaches 391.55 °C, and 200 ppm yields 385.70 °C. The maintained or even elevated EGT levels in HNCB blends under high EGR suggest enhanced combustion resilience in oxygen-deficient environments. For mid-load conditions (12–18 Nm), D + E + HNCB (100 ppm) and (200 ppm) continue to produce higher EGTs than diesel, pointing to more complete combustion and better thermal conversion. For example, at 18 Nm and 20% EGR, D + E + HNCB (100 ppm) reaches 305.08 °C compared with diesel at 342.52 °C, narrowing the thermal gap created by EGR-induced cooling. It’s also notable that at low torque, HNCB’s effect on EGT is more prominent. At 6 Nm and 20% EGR, EGT reaches 186.64 °C with D + E + HNCB (100 ppm), outperforming diesel (184.50 °C) and D + E (174.50 °C). This suggests that HNCB enhances combustion even under suboptimal thermodynamic conditions.

Ethanol-HNCB additives especially at 100 ppm are effective in maintaining or increasing EGT across all loads and EGR levels, demonstrating their capability to improve combustion completeness and thermal efficiency under both ideal and diluted intake conditions.

**Figure 15:**
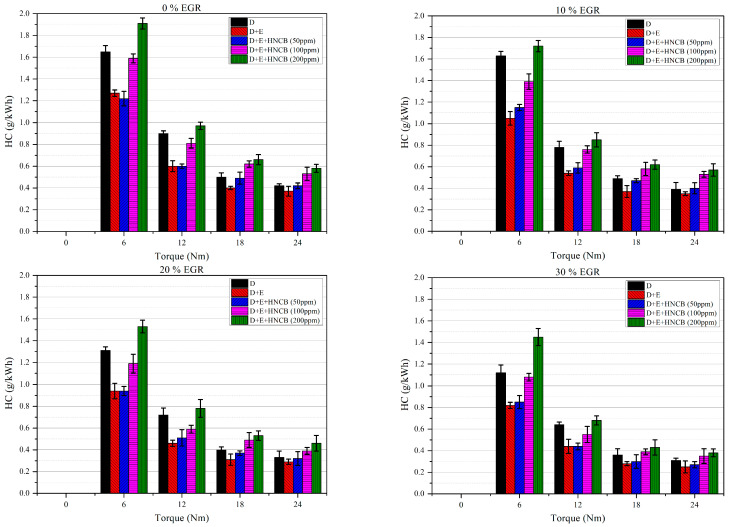
The effects of EGR and ethanol/HNCB additive to diesel fuel on HC emissions. Error bars indicate the estimated measurement uncertainty range of the experimental system.

Figure 15 presents the variation in HC emissions with engine torque, EGR rate, and fuel composition. At 0% EGR, HC emissions decrease markedly with increasing torque for all fuel blends, reflecting improved combustion efficiency at higher loads. Neat diesel exhibits the highest HC levels at low load (6 Nm), reaching approximately 1.65 g/kWh, while the addition of ethanol (D + E) significantly reduces HC emissions across all operating points. This reduction is attributed to enhanced fuel volatility and improved air–fuel mixing promoted by ethanol. The inclusion of HNCB at low concentrations (50–100 ppm) further improves HC performance. For instance, at 12 Nm and 0% EGR, the D + E + HNCB (50 ppm) blend reduces HC emissions to 0.60 g/kWh, compared with 0.90 g/kWh for neat diesel. This indicates that low-dose HNCB enhances ignition stability and flame propagation, particularly under moderate load conditions. Conversely, the 200 ppm HNCB blend consistently produces higher HC emissions, suggesting that excessive additive concentration may delay oxidation reactions or promote locally rich combustion zones. As the EGR rate increases, HC emissions rise for all fuel blends, which is consistent with reduced oxygen availability and lower in-cylinder temperatures caused by exhaust gas dilution. At 30% EGR and 6 Nm, HC emissions increase to 1.12 g/kWh for diesel and up to 1.45 g/kWh for the 200 ppm HNCB blend. This confirms that high EGR levels intensify incomplete combustion, especially when combined with excessive additive concentration. Despite the overall HC-increasing tendency of EGR, ethanol and low-dose HNCB (50–100 ppm) partially mitigate this effect. At 20% EGR and 12 Nm, the D + E + HNCB (50 ppm) blend exhibits HC emissions of 0.51 g/kWh, compared with 0.72 g/kWh for diesel, demonstrating that optimized additive usage can compensate for EGR-induced combustion deterioration. At higher loads (18–24 Nm), HC emissions converge to relatively low values for all fuels, falling below 0.6 g/kWh, even at elevated EGR rates. This behavior highlights the dominant role of improved combustion completeness at higher thermal loads, where the influence of EGR and additives becomes less pronounced. The reduction in HC emissions at low and moderate HNCB concentrations can be attributed to improved ignition quality and flame propagation. The reactive intermediates generated from the decomposition of the thiourea moiety promote oxidation of unburned hydrocarbons. However, at higher concentrations (200 ppm), the additive may introduce diffusion limitations and reduce local oxygen availability, particularly under high EGR conditions, leading to incomplete combustion and increased HC emissions.

Overall, the results indicate that while EGR inherently increases HC emissions due to combustion dilution, the combined use of ethanol and optimally dosed HNCB (50–100 ppm) effectively suppresses HC formation on an energy-specific basis. Low additive concentrations enhance ignition quality and flame development, whereas excessive HNCB addition (200 ppm) deteriorates HC performance, particularly under low-load and high-EGR conditions. These findings confirm that fuel formulation and additive optimization are critical for maintaining low HC emissions in EGR-assisted diesel engine operation, in agreement with previous studies [77,78].

**Figure 16:**
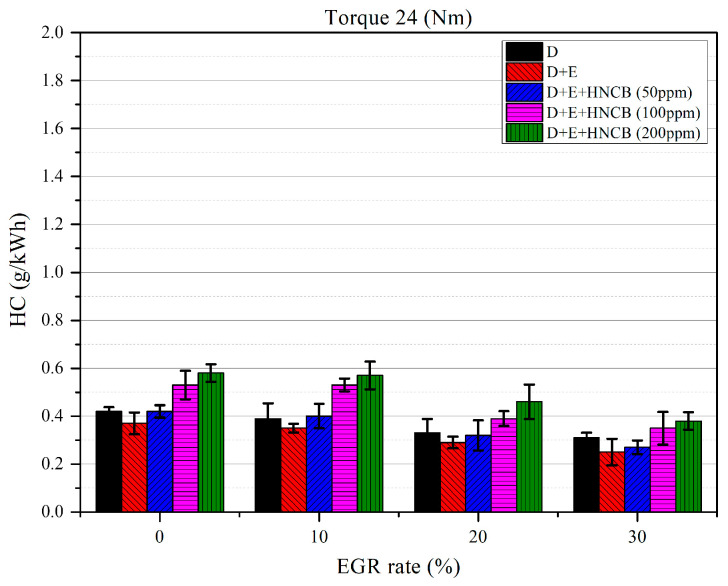
Variation in HC emissions at 24 Nm engine load under different EGR rates (0–30%) for diesel fuel with ethanol and HNCB additive (50–200 ppm). Error bars indicate the estimated measurement uncertainty range of the experimental system.

Figure 16 presents the variation in HC emissions with EGR rate at a constant engine load of 24 Nm. For all fuel blends, HC emissions show a slight but consistent increase with increasing EGR rate, which is attributed to reduced oxygen availability and lower in-cylinder temperatures that promote incomplete oxidation. Neat diesel exhibits HC emissions of approximately 0.42 g/kWh at 0% EGR, decreasing marginally to 0.31 g/kWh at 30% EGR, primarily due to improved combustion efficiency at higher EGR-adapted operating conditions. The D + E blend consistently produces the lowest HC emissions across all EGR rates, highlighting the beneficial effect of ethanol on fuel evaporation and mixture homogeneity. The addition of HNCB at 50 and 100 ppm results in slightly higher HC levels than D + E but remains comparable to or lower than neat diesel, indicating that low-dose HNCB supports stable combustion even under diluted conditions. In contrast, the 200 ppm HNCB blend shows the highest HC emissions at all EGR rates, confirming that excessive additive concentration negatively affects hydrocarbon oxidation, particularly in EGR-assisted combustion.

Overall, the results demonstrate that ethanol and optimally dosed HNCB (50–100 ppm) can effectively limit HC formation at high load, even as EGR rate increases, whereas excessive additive use leads to increased unburned HC emissions.

**Figure 17:**
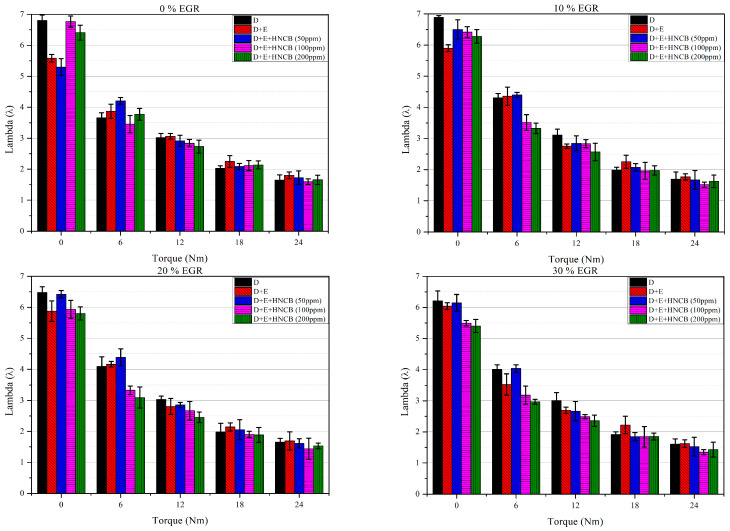
The effects of EGR and ethanol/HNCB additive to diesel fuel on lambda value. Error bars indicate the estimated measurement uncertainty range of the experimental system.

Figure 17 and the accompanying data table present the variation in lambda (λ) values, which were measured using an exhaust gas analyzer under various engine loads and EGR conditions for different fuel blends. The lambda value represents the air–fuel ratio relative to the stoichiometric condition (λ = 1). Values greater than 1 indicate lean mixtures, while values below 1 reflect rich combustion. Lambda was not fixed as an independent control parameter in this study. The experiments were performed at constant engine speed, selected torque levels, and predefined EGR rates, while lambda was measured as a response parameter reflecting the combined effects of fuel formulation, EGR dilution, and combustion behavior. Fixing the lambda would require additional adjustment of the air or fuel supply and could therefore mask the direct influence of the tested fuel blends.

At 0% EGR, lambda values range from 6.799 for diesel at 0 Nm to 1.645 at 24 Nm, indicating a consistent decrease with increasing engine load due to higher fuel delivery. Among fuel blends, D + E + HNCB (50 ppm) produces the lowest lambda (5.3) at 0 Nm, suggesting richer combustion compared with diesel and other blends. Conversely, the D + E blend generally shows lower λ values than diesel, indicating that ethanol, despite being oxygenated, leads to localized richer zones, particularly at low torque. As EGR increases from 10% to 30%, a general decrease in lambda values is observed across all fuel types and loads. This is attributed to the reduction in oxygen content within the intake charge due to recirculated exhaust gases. For instance, at 30% EGR and 24 Nm, λ drops to 1.605 for diesel and reaches as low as 1.347 for D + E + HNCB (100 ppm). This indicates a shift toward richer combustion, particularly in additive-enhanced blends, which may be due to delayed oxidation and extended combustion duration under EGR dilution. At mid-range torque levels (12–18 Nm), lambda values stabilize around 2.0–3.0 depending on fuel type and EGR rate. D + E + HNCB (100 ppm) consistently yields slightly lower lambda values than diesel, indicating enhanced combustion activity and faster reaction kinetics, likely driven by improved ignition and flame speed due to HNCB. The HNCB additive at 200 ppm, while oxygen-rich, appears to reduce lambda slightly at higher loads and EGR rates. This suggests a richer mixture environment, potentially due to faster heat release and more complete fuel vaporization, which may reduce unburned oxygen in the exhaust.

Lambda values measured via exhaust gas analyzer show that increasing torque and EGR ratio decrease λ, moving combustion toward richer conditions. While ethanol and HNCB additives affect λ moderately, the 100 ppm HNCB blend provides a favorable balance between lean-burn efficiency and stable combustion across a wide operating range.

**Figure 18:**
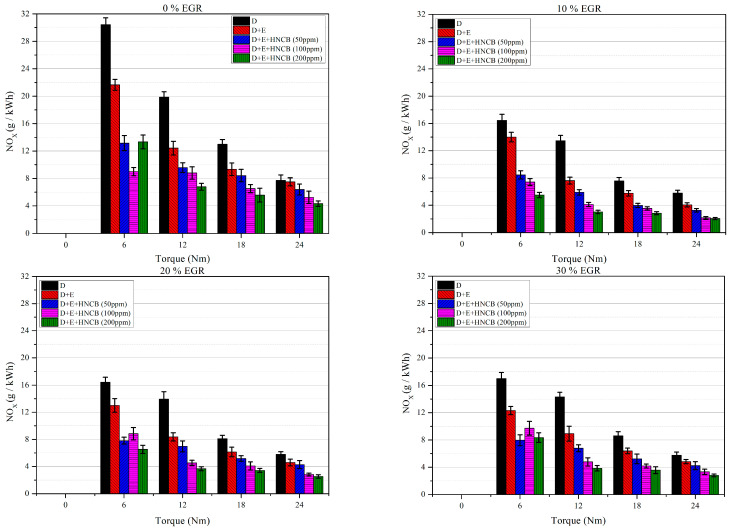
The effects of EGR and ethanol/HNCB additive to diesel fuel on NO_x_ emissions. Error bars indicate the estimated measurement uncertainty range of the experimental system.

Figure 18 illustrates the variations in NO_x_ emissions across different engine torques and EGR rates for various fuel blends, including ethanol and HNCB additives. At low engine loads, relatively high specific NO_x_ values are observed despite moderate concentration levels (ppm). For instance, at 0% EGR and 6 Nm, neat diesel exhibits a NO_x_ emission of 30.43 g/kWh. This behavior is mainly attributed to the low brake power output at light-load conditions, which amplifies the specific emission values. As engine load increases, g/kWh-based NO_x_ emissions decrease consistently for all fuel blends, reflecting improved combustion efficiency and higher effective power output at elevated torque levels. Under 0% EGR conditions, diesel fuel produces the highest NO_x_ emissions across the entire load range. At 18 Nm, diesel reaches 12.99 g/kWh, whereas the D + E blend reduces this value to 9.35 g/kWh. The introduction of HNCB leads to a more pronounced reduction: NO_x_ emissions decrease to 6.52 g/kWh and 5.57 g/kWh for 100 ppm and 200 ppm HNCB, respectively. These results demonstrate that even in the absence of EGR, the combined effects of ethanol’s latent heat of vaporization and the combustion-modifying characteristics of HNCB significantly suppress thermal NO_x_ formation. As EGR is applied, a substantial reduction in specific NO_x_ emissions is observed for all fuel blends. At 10% EGR and 24 Nm, diesel emits 5.79 g/kWh of NO_x_, while the D + E blend reduces this value to 4.06 g/kWh. The addition of HNCB further enhances this effect, resulting in NO_x_ emissions of 3.26 g/kWh, 2.16 g/kWh, and 2.04 g/kWh for 50 ppm, 100 ppm, and 200 ppm HNCB, respectively. Compared with neat diesel, the D + E + HNCB (200 ppm) blend achieves an approximate 65% reduction in specific NO_x_ emissions at this operating condition, highlighting the strong synergistic interaction between EGR and the additive. Similar trends are observed at higher EGR ratios. At 20% and 30% EGR, NO_x_ emissions continue to decline across all loads, with HNCB-added blends consistently exhibiting the lowest g/kWh values. At 30% EGR and 24 Nm, diesel produces 5.74 g/kWh, whereas the D + E + HNCB (200 ppm) blend limits NO_x_ emissions to 2.80 g/kWh. These results confirm that increasing EGR rates effectively reduce oxygen availability and peak combustion temperatures, while the presence of ethanol and HNCB further moderates combustion kinetics and temperature gradients, thereby restricting thermal NO_x_ pathways. The significant reduction in NO_x_ emissions with HNCB addition can be explained by both thermal and chemical effects. The presence of nitrogen-containing functional groups in HNCB may influence the radical pool, promoting pathways that compete with NO formation reactions. Additionally, the enhanced combustion efficiency at moderate additive concentrations reduces localized high-temperature zones, thereby suppressing thermal NO_x_ formation via the Zeldovich mechanism. The synergistic interaction with EGR further amplifies this effect by lowering peak combustion temperatures and oxygen concentration. At higher concentrations, the additive may also contribute to heat absorption during decomposition, further reducing flame temperature and NO_x_ formation.

Overall, the g/kWh-based evaluation confirms that ethanol and HNCB additives not only reduce NO_x_ formation through temperature moderation and improved combustion characteristics, but also effectively enhance the NO_x_-reducing role of EGR, especially at medium and high engine loads. These findings are in good agreement with previous studies reported in the literature, which emphasize the synergistic benefits of oxygenated fuels, combustion-modifying additives, and EGR for achieving cleaner diesel engine operation [79,80].

**Figure 19:**
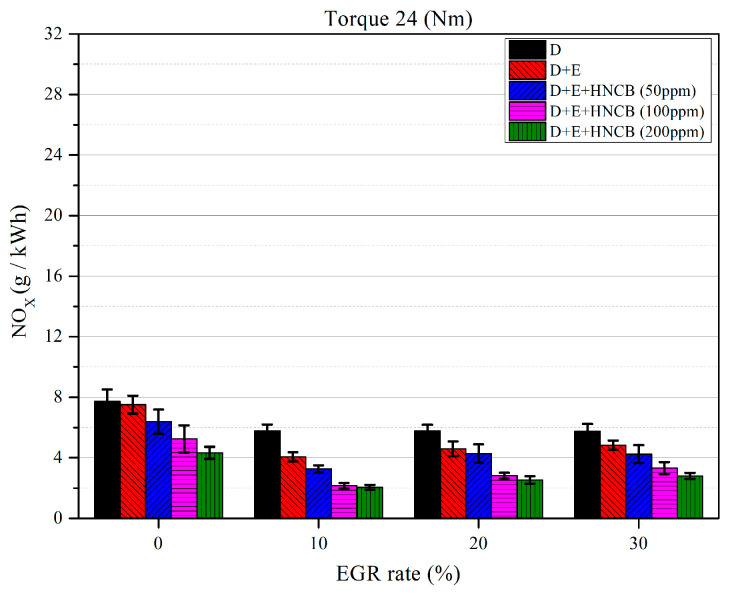
Variation in NO_x_ emissions at 24 Nm engine load under different EGR rates (0–30%) for diesel fuel with ethanol and HNCB additive (50–200 ppm). Error bars indicate the estimated measurement uncertainty range of the experimental system.

Figure 19 shows the variation in brake-specific NO_x_ emissions (g/kWh) with EGR rate at a constant engine load of 24 Nm for different fuel blends. For all fuels, increasing the EGR rate from 0% to 10% results in a marked reduction in NO_x_ emissions, confirming the effectiveness of EGR in suppressing thermal NO_x_ formation through reduced combustion temperature and oxygen availability. At 10% EGR, the lowest NO_x_ emissions are generally observed. In particular, the D + E + HNCB (200 ppm) blend achieves approximately 2.04 g/kWh, corresponding to nearly a 65% reduction compared with neat diesel at the same load. When the EGR rate is further increased to 20% and 30%, NO_x_ emissions tend to stabilize or slightly increase, likely due to deteriorated combustion conditions at excessive EGR levels. Overall, the results indicate that moderate EGR rates combined with ethanol and HNCB additives provide an effective strategy for NO_x_ reduction at high engine load.

**Figure 20:**
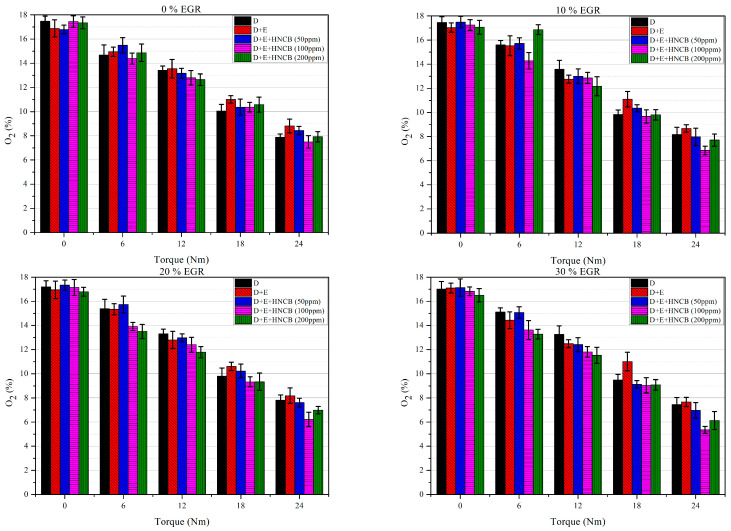
The effects of EGR and ethanol/HNCB additive to diesel fuel on O_2_ emissions. Error bars indicate the estimated measurement uncertainty range of the experimental system.

Figure 20 depicts the effects of various fuel blends (Diesel, Diesel + Ethanol, and Diesel + Ethanol with HNCB additive at 50 ppm, 100 ppm, and 200 ppm) on O_2_ emissions under different torque levels (0–24 Nm) and EGR rates (0%, 10%, 20%, and 30%). Overall, O_2_ concentration in the exhaust decreases as engine load increases across all fuel types. This trend is attributed to the enrichment of the air–fuel mixture under higher load conditions, where more fuel is combusted, leaving less residual oxygen in the exhaust. Under 0% EGR conditions, O_2_ emissions for pure diesel drop from 17.48% at 0 Nm to 7.88% at 24 Nm. When ethanol is added (D + E), slightly higher residual O_2_ levels are observed due to the oxygenated nature of ethanol, which promotes leaner local mixtures rather than complete oxygen consumption. However, the addition of HNCB at 100 ppm and 200 ppm reduces this value again, with O_2_ dropping to 7.5% at 24 Nm, indicating that the additive enhances combustion efficiency and leads to more complete oxygen consumption. At 10% EGR, a similar trend is observed. At 24 Nm, the O_2_ concentration for diesel is 8.17%, whereas for the D + E + HNCB (100 ppm) blend it drops to 6.85%, representing a reduction of approximately 16.2% compared with diesel. The decrease in O_2_ levels is also influenced by EGR, which introduces inert gases such as nitrogen and carbon dioxide into the intake, thus diluting the oxygen concentration. At 20% EGR, O_2_ emissions for diesel are recorded at 7.81% at 24 Nm, while the D + E + HNCB (100 ppm) blend yields a significantly lower value of 6.22%, corresponding to a 20.3% reduction. This further confirms that HNCB additive improves combustion efficiency, particularly at higher loads. Under 30% EGR conditions, the lowest oxygen values are observed. At 24 Nm, the O_2_ level for diesel is 7.46%, while it decreases to 5.36% for the D + E + HNCB (100 ppm) blend, reflecting a 28.1% decrease compared with diesel. This demonstrates that the additive becomes more effective at higher EGR rates, leading to a more complete combustion process. In conclusion, O_2_ emissions decrease significantly with increasing engine load and EGR ratio. While ethanol tends to increase residual oxygen due to its oxygenated nature, the HNCB additive reverses this effect and contributes to more efficient combustion. Notably, the 100 ppm and 200 ppm HNCB doses result in substantial oxygen consumption, yielding lower exhaust O_2_ levels and indicating improved combustion quality.

**Figure 21:**
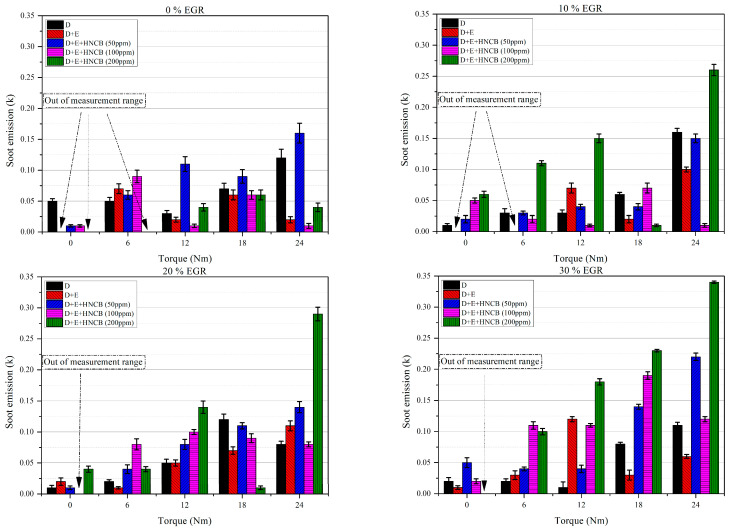
The effects of EGR and ethanol/HNCB additive to diesel fuel on soot emissions. Error bars indicate the estimated measurement uncertainty range of the experimental system.

Soot emissions, measured directly using an exhaust gas analyzer, exhibited considerable variation depending on the fuel blend and EGR rate. As shown in Figure 21, baseline diesel (D) combustion produced relatively higher soot levels, particularly at higher torque loads, due to its rich fuel–air mixture and slower oxidation rate.

The addition of ethanol (D + E) slightly reduced soot formation at lower EGR levels, likely due to the oxygen content of ethanol enhancing the local combustion environment. However, this reduction was marginal and became negligible or inconsistent at higher EGR rates, especially under high torque loads. The inclusion of the HNCB additive demonstrated a more pronounced impact. Specifically, the 50 ppm concentration of HNCB resulted in notably higher soot emissions at full load (24 Nm) and higher EGR settings. This could be attributed to altered spray–combustion interaction or delayed soot oxidation under oxygen-deficient conditions. On the contrary, the 100 ppm and 200 ppm HNCB blends yielded a more balanced behavior. In many cases, 100 ppm provided a moderate reduction in soot, whereas 200 ppm often resulted in either similar or increased soot levels compared with diesel–ethanol blends without additives. Most notably, under 30% EGR and full load, soot emissions for the D + E + HNCB (200 ppm) configuration reached a maximum of 0.34 (k), which was significantly higher than other configurations. This suggests that although HNCB at high concentrations may aid in ignition and HRR control, it may also lead to incomplete oxidation phases in dense exhaust environments. While low-dose HNCB additives may slightly suppress soot emissions under certain conditions, their effectiveness diminishes under high EGR and torque due to rich combustion zones and potential additive-induced particulate formation. These trends emphasize the need for optimized dosing strategies and further investigation into additive combustion chemistry [81,82]. The effect of HNCB on soot formation is governed by competing mechanisms. At moderate concentrations, improved combustion efficiency and enhanced oxidation reduce soot precursors. However, the aromatic nature of HNCB and its relatively high carbon content may contribute to soot formation under oxygen-deficient conditions. At high additive concentrations and elevated EGR rates, incomplete oxidation and rich local zones favor soot nucleation and growth, leading to increased particulate emissions.

Similar reductions in NO_x_ and soot emissions under moderate EGR rates combined with oxygenated additives have been reported in previous diesel engine studies, where enhanced oxidation and reduced peak flame temperatures were observed [69,70]. Overall, the results demonstrate that while moderate HNCB dosing (around 100 ppm) can mitigate soot formation at intermediate EGR rates, excessive additive concentration and high EGR levels lead to a pronounced increase in soot emissions.

**Figure 22:**
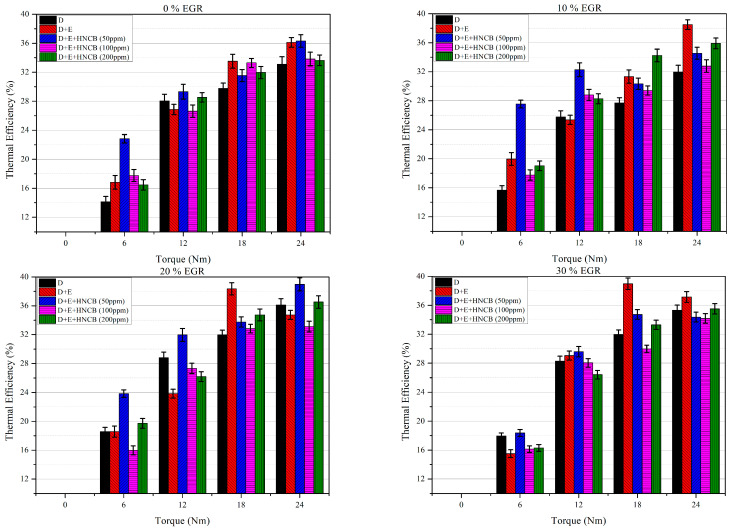
The effects of EGR and ethanol/HNCB additive to diesel fuel on thermal efficiency. Error bars indicate the estimated measurement uncertainty range of the experimental system.

As illustrated in Figure 22, thermal efficiency increased monotonically with engine load for all fuel blends and EGR conditions, mainly due to improved combustion efficiency at higher in-cylinder temperatures and reduced relative heat losses at elevated mean effective pressures. The thermal efficiency reached its maximum at 24 Nm for all cases. The incorporation of the HNCB additive significantly enhanced thermal efficiency compared with neat diesel (D) and the diesel–ethanol blend (D + E), with the most pronounced improvement observed at the 50 ppm concentration. At 0% EGR, the D + E + HNCB (50 ppm) blend exhibited superior performance, particularly at low and medium loads. For example, at 6 Nm, thermal efficiency increased to approximately 23%, compared with about 14% for diesel and 16% for D + E. This improvement may be attributed to micro-explosion–like behavior and/or the intrinsic oxygen supplied by the nitro (–NO_2_) and hydroxyl (–OH) functional groups, which enhance air–fuel mixing under low-load conditions. In contrast, higher additive dosages (100 and 200 ppm) occasionally resulted in reduced efficiency, possibly due to increased fuel viscosity and/or additive agglomeration adversely affecting spray atomization. Under EGR operation, the beneficial effect of the HNCB additive became more evident. At 10% and 20% EGR, the 50 ppm blend consistently delivered the highest thermal efficiency, reaching approximately 39% at 24 Nm under 20% EGR, indicating improved tolerance to charge dilution. At 30% EGR, although all additive blends performed adequately, the D + E blend exhibited relatively stable behavior at higher loads. The improvement in thermal efficiency with HNCB addition, particularly at 50 ppm, can be attributed to optimized combustion phasing and enhanced heat release efficiency. The additive promotes more effective energy conversion by improving ignition timing and reducing combustion losses. However, at higher concentrations, negative effects such as increased viscosity, poor atomization, and delayed combustion may offset these benefits.

Overall, the results demonstrate that HNCB acts as an effective combustion enhancer, with 50 ppm identified as the optimal concentration for maximizing thermal efficiency, particularly under EGR reminder conditions.

**Figure 23:**
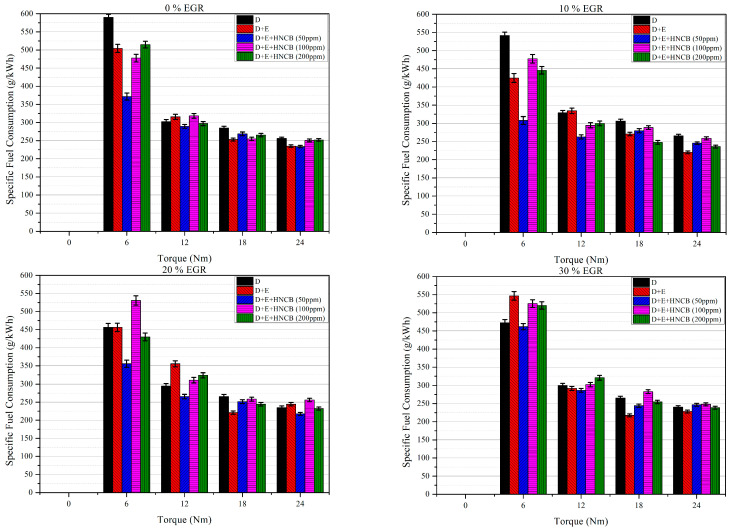
The effects of EGR and ethanol/HNCB additive to diesel fuel on SFC. Error bars indicate the estimated measurement uncertainty range of the experimental system.

As depicted in Figure 23, SFC decreased consistently with increasing engine load for all fuel blends and EGR conditions. This behavior is mainly associated with improved combustion efficiency at higher in-cylinder temperatures and reduced relative heat losses per unit power output. The incorporation of the HNCB additive led to a noticeable improvement in fuel economy, particularly at the 50 ppm dosage, which consistently exhibited lower SFC values compared with neat diesel (D) and the diesel–ethanol blend (D + E). The most pronounced improvement was observed at low load (6 Nm), where the 50 ppm HNCB blend significantly reduced SFC relative to diesel operation. At low load conditions (6 Nm), this improvement corresponds to a reduction in SFC on the order of approximately 35–40% compared with neat diesel operation. Although EGR generally increases SFC due to charge dilution and reduced combustion temperature, the 50 ppm HNCB blend showed enhanced tolerance to EGR. At moderate EGR levels (20%), it maintained the lowest SFC across the tested load range. Even under high EGR conditions (30%), the HNCB-doped fuels remained competitive with diesel, indicating improved combustion stability. The reduction in specific fuel consumption observed with HNCB addition is likely associated with improved combustion efficiency and more complete energy release. Enhanced ignition characteristics and better fuel–air mixing reduce unburned fuel losses. However, the magnitude of the observed reduction suggests that additional factors such as measurement uncertainty, fuel property variations, or load-dependent combustion dynamics may also contribute to the results. Therefore, these findings should be interpreted within the context of experimental conditions.

Overall, the results suggest that the HNCB additive, particularly at 50 ppm, enhances fuel utilization efficiency by promoting more effective combustion, especially under low-load and EGR-diluted conditions. Briefly, the observed improvements in thermal efficiency and reductions in specific fuel consumption are consistent with earlier findings reported for oxygenated fuel blends and low-dose combustion-enhancing additives [83,84].

## 4. Conclusions

This study comprehensively investigated the effects of ethanol (E) and combustion enhancer (HNCB) at different concentrations (50, 100, and 200 ppm) blended with diesel fuel (D) on the combustion, performance, and emission characteristics of a single-cylinder diesel engine under four EGR conditions (0%, 10%, 20%, and 30%) and five torque levels (0–24 Nm). The findings provide insightful correlations and reveal the optimal strategies for cleaner and more efficient engine operation.

- The maximum in-cylinder pressure (P_max_) increased with ethanol addition and further improved with the 100 ppm HNCB additive. At 24 Nm torque and 0% EGR, D + E + HNCB (100 ppm) yielded a Pmax increase of 4.1% over neat diesel. MPRR (Maximum Pressure Rise Rate) values followed a similar trend, where the highest increase was recorded with HNCB 100 ppm, especially under higher EGR levels.
- Cyclic variations in IMEP showed a significant reduction with D + E + HNCB, implying more stable combustion. CA10 and CA50 values indicated that ethanol and HNCB led to advanced ignition timing and accelerated combustion phasing, especially evident under low EGR. On the other hand, CA90 values increased with additive concentration, implying delayed combustion completion, particularly for D + E + HNCB (200 ppm).
- Thermal Efficiency exhibited a positive correlation with engine load across all tested fuels. The 50 ppm HNCB concentration emerged as the optimal dosage for thermal conversion efficiency, effectively compensating for the dilution effects of EGR. Specifically, at 20% EGR and high load, the 50 ppm blend outperformed the baseline D + E mixture, suggesting that the oxygenated nitro group within the additive structure enhances the combustion kinetics even in oxygen-deficient environments.
- Regarding fuel economy, the 50 ppm HNCB blend demonstrated superior performance compared with higher concentrations, resulting in a notable reduction in specific fuel consumption (SFC). Most prominently, under low-load conditions (6 Nm) and 0% EGR, the 50 ppm blend exhibited a reduction in SFC of up to approximately 37% relative to neat diesel. This improvement can be attributed to enhanced combustion efficiency associated with improved ignition characteristics and more effective fuel–air mixing at low additive concentrations. The presence of nitrogen- and sulfur-containing functional groups in HNCB may promote the formation of reactive intermediates during the early stages of combustion, thereby facilitating more complete energy release. However, it should be noted that the magnitude of SFC reduction is influenced by operating conditions and measurement sensitivity and therefore should be interpreted within the context of low-load combustion dynamics.
- CO emissions were generally reduced with ethanol and HNCB up to 100 ppm. However, 200 ppm led to marginal increases due to possible over-enrichment and incomplete combustion. HC emissions increased with load and EGR rate but were significantly higher for HNCB (200 ppm), especially at 30% EGR (up to 49 ppm, nearly 75% higher than diesel).
- NO_x_ emissions were considerably lower in D + E + HNCB (100–200 ppm) compared with diesel, particularly under higher EGR, showing reductions of up to 65–75%, indicating effective thermal NO_x_ suppression.
- The lambda values (measured via exhaust gas analyzer) showed a downward trend with load, indicating richer combustion mixtures. HNCB at 100 and 200 ppm slightly enriched the mixture due to enhanced ignition, yet all values remained within stoichiometric range.
- Soot emissions demonstrated a complex behavior. At 24 Nm, D + E reduced soot by 83%, whereas HNCB at 100 ppm showed up to 91% reduction under 0% EGR. However, at 30% EGR, HNCB at 200 ppm increased soot by over 200%, highlighting the critical importance of dosing.
- Ethanol blending (D + E) consistently improved combustion phasing and reduced CO and soot. HNCB at 100 ppm appeared as the most balanced strategy in terms of combustion stability, performance, and emission reduction. Higher doses (200 ppm) were effective at low EGR but led to higher HC and soot emissions at high EGR due to potential overfueling and poor atomization. A 20% EGR with D + E + HNCB (100 ppm) provided the optimal trade-off between NOx reduction, combustion efficiency, and soot suppression.

The combined use of ethanol and HNCB influenced combustion and emission behavior in a load- and EGR-dependent manner, with promising results obtained at selected additive concentrations and operating conditions. Nevertheless, the highly non-linear interaction observed between additive concentration, EGR rate, and engine load indicates that fixed-ratio additive formulations may not yield consistent benefits across all operating regimes. While 100 ppm HNCB emerged as the most balanced dosage, higher concentrations (e.g., 200 ppm) produced adverse effects under oxygen-deficient or high-EGR conditions, emphasizing the need for more adaptive control strategies.

Future studies should therefore focus on developing real-time additive–EGR optimization frameworks, potentially incorporating closed-loop combustion phasing control, variable EGR scheduling, or adaptive injection strategies to maintain favorable in-cylinder thermodynamic conditions. Moreover, future work may investigate the influence of HNCB on the apparent cetane number of fuel blends using standardized CFR engine tests, which are specifically designed for cetane number determination, in order to further correlate observed combustion phasing behavior with conventional ignition quality metrics. Additional attention should also be directed toward long-term durability, injector deposit formation, lubrication impacts, and environmental-toxicity assessments, ensuring that the additive meets emerging sustainability and regulatory criteria. Collectively, such studies will help clarify the broader applicability and limitations of HNCB-derived combustion modifiers in diesel–ethanol systems.

## Figures and Tables

**Table 1:** Test fuels and mixing ratios.

Test Fuels	Mixing Ratios
D	100% Diesel (1000 mL)
D + E	100% Diesel + 10 mL Ethanol
D + E + HNCB (50 ppm)	100% Diesel + 10 mL Ethanol with HNCB (50 ppm)
D + E + HNCB (100 ppm)	100% Diesel + 10 mL Ethanol with HNCB (100 ppm)
D + E + HNCB (200 ppm)	100% Diesel + 10 mL Ethanol with HNCB (200 ppm)

**Table 2:** Test Fuel Properties [52,53,54,55,56].

Properties	Diesel	Ethanol
Chemical Formula	C_12_H_26_-C_14_H_30_	C_2_H_5_OH
Molecular weight	170–198	46
Energy Content (MJ/kg)	42.600	26.700
Flash Point (°C)	70	13
Boiling Point (°C)	180–360	78
Density (kg/m^3^)	846	789
Viscosity (mPa.s)	3.546	1.074
Autoignition Temperature (°C)	256	420
Cetane Number	40–55	0–5

**Table 3:** Test engine specifications.

Parameter	Specification/Value
Brand&Model	Lombardini 3LD510
Engine type	Single cylinder, DI
Cooling system	Air cooling
Max power	12 Hp @ 3000 rpm
Max torque	32 Nm @ 1800 rpm
Cylinder volume	510 (cm^3^)
Compression ratio	17.5:1
Bore × Stroke	85 × 90 (mm × mm)
Fuel	Diesel
Injection pressure	190–200 (bar)
Number of injection nozzle	4
Injection timing (start and stop)	705° and 729° CA
Injection spray angle	160°
Intake valve opening (IVO)	16° BTDC
Intake valve closing (IVC)	40° ABDC
Exhaust valve opening (EVO)	40° BBDC
Exhaust valve closing (EVC)	16° ATDC

**Table 4:** Instrument uncertainties used in the present experiments.

Parameter	Instrument	Range	Uncertainty
Engine speed	Optical tachometer	0–5000 rpm	±1 rpm
Torque	Eddy-current dynamometer	0–50 Nm	±0.2 Nm
Fuel consumption	Gravimetric balance	0–5 kg/h	±0.5%
Exhaust gas temperature	K-type thermocouple	0–1000 °C	±1 °C
In-cylinder pressure	Piezoelectric sensor	0–250 bar	±1%
Crank angle	Optical encoder	0–720 °CA	±0.1 °CA
CO, CO_2_	Gas analyzer	-	±0.2 vol.%
HC	Gas analyzer	-	±5 ppm
NO_x_	Gas analyzer	-	±5 ppm
Smoke opacity	Opacimeter	0–100%	±1%

## Data Availability

The raw data supporting the conclusions of this article will be made available by the author on request.

## Abbreviations

The following abbreviations are used in this manuscript:

ABDC	After bottom dead center
ATDC	After top dead center
BBDC	Before bottom dead center
BTDC	Before top dead center
BTE	Brake thermal efficiency
CA5	Crank angle at 5% cumulative heat release
CA10	Crank angle at 10% cumulative heat release
CA50	Crank angle at 50% cumulative heat release
CA90	Crank angle at 90% cumulative heat release
CHR	Cumulative heat release
CI	Compression ignition
CO	Carbon monoxide
CO	Carbon dioxide
COV_IMEP	Coefficient of variation of indicated mean effective pressure
D	Diesel fuel
D + E	Diesel–ethanol blend
EGR	Exhaust gas recirculation
EGT	Exhaust gas temperature
EVC	Exhaust valve closing
EVO	Exhaust valve opening
FT-IR	Fourier-transform infrared spectroscopy
GUM	Guide to the Expression of Uncertainty in Measurement
HC	Hydrocarbons
HNCB	2-chloro-N-((2-hydroxy-4-nitrophenyl)carbamothioyl)benzamide
HRR	Heat release rate
ICE	Internal combustion engine
IMEP	Indicated mean effective pressure
IVC	Intake valve closing
IVO	Intake valve opening
KSCN	Potassium thiocyanate
LHV	Lower heating value
MPRR	Maximum pressure rise rate
NMR	Nuclear magnetic resonance
NO_x_	Nitrogen oxides
O_2_	Oxygen
PM	Particulate matter
SFC	Specific fuel consumption
TDC	Top dead center
